# Seasonal Patterns of Hormones, Macroparasites, and Microparasites in Wild African Ungulates: The Interplay among Stress, Reproduction, and Disease

**DOI:** 10.1371/journal.pone.0120800

**Published:** 2015-04-15

**Authors:** Carrie A. Cizauskas, Wendy C. Turner, Neville Pitts, Wayne M. Getz

**Affiliations:** 1 Department of Environmental Science, Policy, and Management, University of California, Berkeley, CA, United States of America; 2 Department of Ecology and Evolutionary Biology, Princeton University, Princeton, NJ, United States of America; 3 Centre for Ecological and Evolutionary Synthesis, Department of Biosciences, University of Oslo, Oslo, Norway; 4 School of Physiology, University of the Witwatersrand, Johannesburg, South Africa; 5 School of Mathematical Sciences, University of KwaZulu-Natal, Durban, South Africa; University of Minnesota, UNITED STATES

## Abstract

Sex hormones, reproductive status, and pathogen load all affect stress. Together with stress, these factors can modulate the immune system and affect disease incidence. Thus, it is important to concurrently measure these factors, along with their seasonal fluctuations, to better understand their complex interactions. Using steroid hormone metabolites from fecal samples, we examined seasonal correlations among zebra and springbok stress, reproduction, gastrointestinal (GI) parasite infections, and anthrax infection signatures in zebra and springbok in Etosha National Park (ENP), Namibia, and found strong seasonal effects. Infection intensities of all three GI macroparasites examined (strongyle helminths, *Strongyloides* helminths, and *Eimeria* coccidia) were highest in the wet season, concurrent with the timing of anthrax outbreaks. Parasites also declined with increased acquired immune responses. We found hormonal evidence that both mares and ewes are overwhelmingly seasonal breeders in ENP, and that reproductive hormones are correlated with immunosuppression and higher susceptibility to GI parasite infections. Stress hormones largely peak in the dry season, particularly in zebra, when parasite infection intensities are lowest, and are most strongly correlated with host mid-gestation rather than with parasite infection intensity. Given the evidence that GI parasites can cause host pathology, immunomodulation, and immunosuppression, their persistence in ENP hosts without inducing chronic stress responses supports the hypothesis that hosts are tolerant of their parasites. Such tolerance would help to explain the ubiquity of these organisms in ENP herbivores, even in the face of their potential immunomodulatory trade-offs with anti-anthrax immunity.

## Introduction

Stressors can take the form of acute, unpleasant, and usually unpredictable stimuli that precipitate a stress response. The resulting hormonal, physiological, and behavioral changes help animals cope with the stressor and enhance survival [1,2]. Glucocorticoids (GCs), adrenal hormones of the hypothalamic-pituitary-adrenal (HPA) axis that are the primary mediators of the stress response, are helpful during acute stressors but can be problematic when elevated for several hours a day over weeks to months [1]. This chronic increase in GCs has several negative physiological effects, including metabolic disruption, reproductive suppression, immunosuppression and immunomodulation, and increased susceptibility to disease [3–5].

Pathogens themselves can also cause chronic stress in their hosts. While evidence for the ability of macroparasites to cause host stress is equivocal, several studies have found significant, positive relationships between parasite infection intensity or prevalence and GC concentration (e.g. [6,7]). Common gastrointestinal (GI) nematode parasites, such as strongyle and *Strongyloides* helminths, can have significant negative fitness impacts on hosts, such as impairing growth and causing weight loss, undernutrition, and reduced fecundity [8,9]. Coccidian GI parasites can negatively affect total body mass, impair growth and food utilization, cause epithelial cell damage and inflammation, and result in diarrhea, weight loss, and even death [9,10]. This interplay between stress and disease, however—particularly between stress and macroparasites—has been understudied in natural systems and is poorly understood.

Etosha National Park (ENP), Namibia, is a highly seasonal environment; it experiences one rainy season for approximately three to four months per year, followed by a cold dry and then a hot dry season [11,12]. Two of the predominant plains ungulates in ENP, plains zebra (*Equus quagga*) and springbok (*Antidorcas marsupialis*) are affected by these seasonal changes. While there is nearly 100% prevalence of strongyle nematodes in zebra in this system year-round, zebra experience a significant increase in strongyle infection intensity in the wet season compared to in the dry [13]. Springbok in ENP also experience significantly greater infection intensity in three gastrointestinal parasites (strongyle nematodes, *Strongyloides* nematodes, and *Eimeria* coccidia) during the wet season [13]. Zebra and springbok also experience annual anthrax outbreaks in the wet season, caused by the bacterium *Bacillus anthracis* [14]; during this season, it has been estimated that up to 400 zebras die of anthrax, out of a population of approximately 13,000 animals (estimations of springbok anthrax mortality have not been conducted using these methods, but springbok and zebra are the primary anthrax hosts in ENP) [15]. ENP anthrax outbreaks occur shortly after parasite infection intensities peak [16,17], and we have found that GI parasites may contribute to increased zebra susceptibility to this pathogen at this time due to immunomodulatory effects [18].

Hosts can experience other seasonally-driven potential stressors as well. Dry seasons often bring nutritional depletion [17], potentially leading to increased GC secretion [19,20]. While both zebra and springbok are not strictly seasonal breeders, both typically give birth during the wet season; zebra often mate again soon thereafter, while springbok often mate in the cold dry season (July). Thus, most mares and ewes experience mid-gestation during the hot dry season [11,13,21–23]. Reproductive behaviors such as rutting [24], and gestation and lactation [25] are particularly energy demanding, and most last for an extended period, potentially leading to chronic stress [26]. Reproductive hormones themselves can also be immunosuppressive, potentially influencing susceptibility to disease [27].

While studies have examined the impact of chronic stress in wildlife and the interaction between stress and seasonality, or reproduction, or disease individually (e.g. [26,28–30], we are unaware of any that have examined the seasonality of stress responses in combination with environmental changes, reproductive efforts, pathogen pressures and interactions, and immune function. Because all of these factors are closely interlinked, it is important that studies begin examining this complex array of factors in concert with each other. This study examines the seasonal fluctuations in stress hormones, in conjunction with environmental parameters, reproductive and gestational status, and pathogen interactions in zebra and springbok in ENP. As there is such strong seasonality in this system for most parameters examined, we hypothesized that stress responses would be significantly higher in the wet season compared to in the dry season: zebra appear to engage in most of their intense reproductive behaviors in the wet season (birth, lactation, estrus), springbok largely birth early in and lactate through the wet season, and both zebra and springbok experience significantly higher infection intensities of macroparasite and microparasite infections during this time. In addition, because we have previously found that zebra GI parasites likely exert more selection pressure than do the other pathogens in this system [31], and that they influence host immunity during peak infection intensities [18], we hypothesized that high levels of parasitism would correspond most strongly with stress hormone responses out of all the factors examined. Understanding the primary drivers of host stress is important for determining the full extent of pathogen effects, as well as for determining potential avenues for intervention in conservation programs.

## Methods

### Study Area and Species

Etosha National Park (ENP) is a 22,915 km^2^ fenced conservation area in northern Namibia, located between 18°30’S-19°30’S and 14°15’E-17°10’E (Fig. A in S1 File). Rainfall in ENP is highly seasonal: the rainy season lasts from December through April, with the greatest rainfall occurring during January and February, and 80% of all rain falling between December and March [11,32] (Fig. B in S1 File). The only perennial water available to the park’s wildlife is found in man-made boreholes, or in natural artesian or contact springs [33]. Permits for this research were issued by the Namibian Ministry of Environment and Tourism (MET) and the Etosha Ecological Institute (EEI).

Plains zebra (*Equus quagga*) and springbok (*Antidorcas marsupialis*; members of the family Bovidae) are the two most abundant plains ungulate species in ENP, with populations fluctuating around 13,000 (95% CI rounded to nearest 100: 10,900–15,000) and 15,600 (13,200–17,900) (EEI unpublished aerial survey data 2005), though these numbers are likely an underestimate for springbok. Neither of the species involved in this research is endangered nor protected.

### Zebra Capture and Sampling

We obtained whole blood, serum, fecal, and ectoparasite samples from collared zebra over five seasons between 2008 and 2010 (Table A in S1 File). We immobilized, sampled, and released all animals safely under animal handling protocol AUP R217-0509B (University of California, Berkeley). A Namibian Ministry of Environment and Tourism veterinarian was present for all captures, as was author Cizauskas, who is also a licensed veterinarian. Animals were anesthetized for a short time (5–10 minutes), using a combination of etorphine, azaperone, and detomidine delivered via Pneu-Darts (Williamsport, PA). Anesthesia was monitored in the field and animals were given reversal medication at the end of sample collection and observed until they awoke and moved off normally. The work in Etosha National Park, Namibia, was approved by the Namibian Ministry of Environment and Tourism (MET) and the Etosha Ecological Institute (EEI). We originally sampled 38 animals in the wet season, intending to recapture them as many times as possible in five subsequent seasons roughly six months apart to both control for individual variation (Table A in S1 File). If we could not re-capture all initially collared individuals, we captured and collared new individuals in order to have a similar sample size for each season. We conducted a total of 144 zebra capture events; all captured animals for this study were adult females to control for sex differences. We further determined age to half a year by combining tooth eruption observations, caliper measurements of upper incisors, and patterns of wear, and adding on the time between subsequent captures and the time of first dental evaluation for each individual [34]. We recorded, when possible, whether a mare was actively caring for a foal (0 for no foal; 1 for foal present); whether a mare was pregnant (0 for not pregnant; 1 for possibly pregnant based on palpation and visual determination; and 2 for definitely pregnant); and whether a mare was lactating (0 for no lactation; 1 for watery, straw-colored liquid able to be expressed from teats; 2 for milk able to be expressed from teats).

We sampled 69 individuals overall, with 20 sampled twice, 11 sampled three times, 12 sampled four times, and two sampled five times (Table B in S1 File). We collected blood from peripheral veins for blood smears and hematocrit (HCT) analysis, and removed serum for antibody analyses. From blood smears we obtained neutrophil and lymphocyte counts (S1 File). Neutrophils are white blood cells instrumental in the innate immune and inflammatory responses against primarily extracellular pathogens and possibly against certain intracellular bacteria [35]. Lymphocytes include the different types of T and B cells that are activated in an antigen-specific manner as components of the adaptive immune system [36].

From serum we measured anti-anthrax antibody titers using enzyme-linked immunosorbent assays (ELISAs) (S1 File). Antibodies against the protective antigen (PA) toxin component of anthrax have been shown to be essential for adaptive protection against anthrax [37,38]. As a bacterial infection, anthrax provokes primarily a Th1-type immune response, in contrast to the Th2-type response involved in macroparasite infections [39]. We used an ELISA procedure with wildtype *Bacillus anthracis* PA as coating antigen to measure host anti-PA titers ([40]; S1 File).

When possible, we collected feces by observing an individual prior to capture and collecting a homogenized sub-sample within ten minutes of defecation. For animals that were not observed defecating, we collected fecal samples when possible by inserting a gloved hand into the rectum. As capture events took place between 9:00 and 14:00, fecal samples were collected within this same time window, thereby controlling for potential differences in timing of fecal egg shedding [41].

We collected and counted all visible ectoparasites, regardless of life stage, on zebras during capture events. The vast majority of ticks observed were *Rhipicephalus evertsi mimeticus*, a tick species found throughout Namibia in wild equids and greater kudu [42]. These ticks parasitize hosts year-round, with more adults present from November to May and immature stages peaking from February to March and May to September [43].

### Non-Captured Zebra and Springbok Sampling

Fecal samples were collected noninvasively from zebra and springbok in the Okaukuejo area approximately monthly between August 2005 and May 2007 [12,13]. Fecal collection took place in the first week of each month, with a mean of 37 and 35 samples collected per month for zebra and springbok, respectively. Samples were collected within 100m of roads and between 7:00 and 13:00. For each fecal sample, date, time, species, sex, and age category were recorded. Age and sex were determined within one of three age classes: juveniles for those <1 year old; yearlings for those 1–2 years of age; and adults for those 2+ years old using physical characteristics as previously described [13]. Samples were refrigerated immediately and evaluated for GI parasites within 48hrs, after which they were frozen until hormone extraction (within 18 months of sampling).

We subsampled this large sample set for hormone analyses. We selected only yearlings and adults to control for effects of sexual maturity or near sexual maturity on hormone profiles. We selected male and female samples, and zebra and springbok samples in approximately equal numbers. We chose samples representing similar seasons to those of captured zebra samplings: February-May for the "wet" season, and August-October for the "dry" season. Ultimately, 318 zebra (22 male yearlings, 11 female yearlings, 136 male adults, and 149 female adults) and 272 springbok extracted samples (22 male yearlings, 35 female yearlings, 106 male adults, and 107 female adults) were used for hormone analysis.

### Rainfall Quantification

Previous studies in this system determined that helminth infection intensity in zebras and springbok is significantly related to rainfall one and two months prior, with both strongly correlated with helminth eggs and coccidian oocysts shed per gram of feces [12]. We determined cumulative rainfall over the two months prior to sampling by adding up daily rainfall amounts (in mm) over the 60 days prior to each individual sampling event. We linked each fecal sample to daily rainfall records from the closes management station to the sampling location. For some analyses, we also grouped individuals into wet or dry season categories, based on rainfall quantification (see Supplementary Methods, “Rain Group Determination,” and Fig. B in S1 File).

### Gastrointestinal Parasite Species and Quantification

The gastrointestinal (GI) nematodes examined in these host species were strongyle helminth parasites (abbreviated in models and figures as GIP), *Strongyloides* helminths (abbreviated as GIS), and *Eimeria* coccidia (abbreviated as GIC) (S1 File). Zebra experience infections with strongyles, while springbok experience infections with all three parasite types [13]. All of these parasites exhibit direct life cycles with free-living infectious stages that require 1–2 weeks of development in the external environment prior to becoming infectious to new hosts. These environmental stages are highly susceptible to desiccation [44,45]; thus, it is unsurprising that previous studies in ENP found a strongly seasonal pattern in GI parasite infection intensities, with hosts exhibiting greater new infections during the wet season than in the dry [13].

We evaluated all fecal samples for strongyle eggs, and springbok samples also for *Strongyloides* eggs and *Eimeria* oocysts using a modified McMaster technique for fecal egg counts [46], a commonly used non-invasive method for quantifying parasitism [47] (S1 File).

### Hormone Metabolite Extraction and Measurement

Steroid hormones can be detected in blood and their metabolites can be detected in feces; however, measurements of hormone metabolites in feces are often best in wildlife because fecal collection can often be done noninvasively and is free from stress hormone feedback mechanisms, and fecal samples reflect longer term patterns of hormone trends, dampening potential acute stressor responses or diurnal variations in hormone levels [48,49]. Fecal assays for concentrations of stress and reproductive hormone metabolites have been developed and used extensively in wildlife (e.g. [50,51]). Fecal hormone metabolite concentrations reflect the state of circulating hormones an individual experienced approximately 10–12 hours prior for most ruminants [52], or approximately one day prior for equids [53]. Elevations in hormone levels were interpreted as reflecting host physiological status over at least the previous week; the probability that we collected a sample exactly 12–24 hours after an animal had experienced an acute, transient hormone fluctuation was extremely low, and thus we made the assumption that elevations in hormone metabolites reflected a longer physiological state.

We extracted fecal hormone metabolites from samples using methods modified from a previous similar study [54] (S1 File). We conducted radioimmunoassays for fecal glucocorticoid metabolites (FGM), fecal progestin metabolites (FPM), fecal estrogen metabolites (FEM), and fecal testosterone metabolites (FTM). Hereafter, these will be referred to simply as stress hormones, progesterone, estrogen, and testosterone or by the acronyms listed above. Commercially available kits were used to conduct these assays, and assays were validated for each species and each hormone (S1 File).

### Statistical Analyses

#### Multiple Imputation of Missing Data—Captured Zebras

Imputation is a method of replacing missing observations with plausible estimates based on available data. Multiple imputation methods are particularly useful for imputing missing multivariate data, and rely on available data from multiple predictors and covariates to create a set of datasets for each missing value [55,56]. This method, unlike single imputation methods, provides a variance of an estimate and accounts for uncertainty, given that the value in question was imputed rather than observed [57,58]. While multiple imputation is common in public health research, it has rarely thus far been used in ecological studies.

We imputed missing values from an individual capture event (e.g. we imputed a missing value for estrogen concentration for an individual if the majority of other values were present for that individual at that time); however, if an animal was not captured during one season, we did not impute missing values for that individual in that season. We imputed two missing values for HCT (1.4% of total HCT data), 15 (9.7%) for ectoparasite count, 33 (22.9%) for GIP, 47 (32.6%) for FGM, 59 (40.9%) for FPM, 53 (36.8%) for FEM, 16 (11.1%) for presence or absence of a foal, 21 (14.6%) for pregnancy status, and 16 (11.1%) for lactation status. Few of these missing variables were overlapping for the same individual-capture; thus, discarding cases with imputed data would have resulted in disregarding upwards of 90% of the capture events in our analyses, thereby discarding all the valuable information associated with individuals that had some missing data.

For imputation, we used the Multiple Imputation by Chained Equations (MICE) method with the 'mice' package [56] in R v2.15.2 [59]. We performed 100 imputation cycles and generated five imputations. We averaged the five estimates for each data point to produce single mean estimates for use in *t* tests, while we used the multiply imputed datasets directly in our generalized estimating equations and then determined mean estimates for the model parameters [60]. We adjusted standard errors, Wald statistics, and *p* values according to Rubin's rules [61]. See S1 File for a further discussion of multiple imputation and methods used.

We did not impute missing data for non-captured (NC) zebra or for springbok analyses, due to both the large number of missing hormone measurements and the fact that most missing variables were missing for the same individuals and/or same seasons. For NC zebra, there were 5 missing FGM (1.6% of total FGM data), 92 missing FPM (28.8%), 99 missing FEM (30.9%), and 55 missing FTM measurements (17.2%), nearly all missing from the same 50–100 individuals; for springbok, there were 2 missing FGM (0.73%), 78 missing FPM (28.6%), 102 missing FEM (37.4%), and 51 missing FTM measurements (18.8%), nearly all missing from the same 50–80 individuals. Instead of imputing missing variables, we partitioned these data in subsets based on the parameters of interest and performed several nested analyses of models with decreasing overall sample size (see below).

#### Seasonal Comparisons

For each group of animals examined, we wanted to determine if immune factors, pathogen intensity or exposure, stress hormones, and reproductive hormone levels changed seasonally. We first examined the effects of rainfall for various pathogen and immune factors by comparing data between wet and dry seasons from the first sampling event for each captured zebra. This ensured that we were only comparing unique individuals, and therefore population-level effects, between seasons, without the possibility of autocorrelation and the issue of repeated measures (Table Ba in S1 File). We transformed variables by taking logarithmic, square-root, and fourth-root values to improve assumptions of normality when necessary and possible (Table C in S1 File). We compared normalized GI parasite counts, WBC counts, neutrophil and lymphocyte counts, HCT, and FGM, FEM, and FPM concentrations between seasons using Welch's two-tailed *t* tests. We compared ectoparasite counts and log_2_ anti-PA titers between rain groups using two-sided Wilcoxon rank sum tests. We adjusted *p* values to control for the familywise error rate by using the Holm-Bonferroni method [62]. We then did similar pairwise comparisons across the same animals, between seasons, for first and second captures to examine individual level effects between seasons given individual-level variation that otherwise might have obscured patterns at the population level (Table Bb in S1 File).

Similar to the seasonal comparisons for captured zebra, we performed bulk group comparisons for NC zebra and springbok between rain groups, sexes and age classes. For those response variables that could be successfully transformed to normality (GIP and FTM for NC zebra; FPM, FEM, and FTM for springbok), we performed Type III ANOVAs for unbalanced data to explore seasonal differences and potential interaction effects. We used R v2.15.2 and the 'car' package for these analyses [63]. We used Tukey pairwise post-hoc tests to determine the significance of multiple comparisons. For other response variables that were incapable of being transformed to complete normality, we used Type II ANOVAs and Tukey post-hoc tests to explore potentially significant interaction effects. We then used Wilcoxon rank sum tests to examine rain group, sex, and age differences, and performed these same comparison tests for any interactions significant in the ANOVAs at a *p*≤0.1 level. We adjusted *p* values from Wilcoxon rank sum tests to control for the familywise error rate by using the Holm-Bonferroni method discussed above.

For all GI parasite and FGM comparisons, we included interactions between sex and age, rainfall and sex, and rainfall and age. For FPM, FEM, and FTM, we did not include interactions between sex and age; numbers of yearlings were quite low for this dataset for NC zebras (*N* = 9), and these reproductive hormones were expected to be higher in adults of reproductive age in both species than in yearlings. We did, however, explore interactions between rainfall and sex for FPM and FEM. For FTM, only males were examined. The yearling age group in the NC zebra dataset was also quite low (*N* = 15), so we explored interactions between rainfall and age with caution. The yearling males in the springbok dataset only included 10 individuals, and thus we did not explore rainfall-age interactions for springbok testosterone.

### Coinfections, Immunity, and Hormone Relationships

#### Captured Zebra

We wanted to determine how seasonal factors, hormones, and coinfections affected the prevalence and intensity of each of our pathogens. Thus, we allowed each parasite to play the role of response variable in separate models to avoid biasing the directionality of parasite interactions and to allow each parasite outcome to be examined from the standpoint of potential predisposing immune factors toward that infection alone (Table D in S1 File). For these same reasons, we then used each of our immune parameters and the FGM measurements as the response variable in separate models to allow us to more directly examine the cross-relationships between them and the other physiological parameters and parasites (Table D in S1 File). We did not fit individual models with FPM, FEM, or FTM as response variables because these hormones are more clearly directionally determined by maturity, mating, and pregnancy status. These hormones were measured as means of controlling for underlying seasonal changes in mating and pregnancy, rather than as individual response variables of interest.

We developed generalized estimating equation (GEE) models using R v2.15.2 and the 'geepack' [64] to examine the correlations between pathogen types, immune parameters, and hormone concentrations in captured zebras. In all models we used a working correlation matrix with a first-order autoregressive relationship (AR-1) because, while individual immune, disease, and hormonal factors are likely correlated through time, these correlations should decrease between later time points and earlier samplings [65,66].

We developed each GEE by using a backwards stepwise refinement method based on comparing the quasi-likelihood under the independence model criterion (QIC) values between maximal models and models with variables removed [67]. The QIC is equivalent to Akaike's information criterion (AIC) for GEEs, which are not strictly likelihood based. After stepwise selection of the main terms, we added interaction terms between the remaining explanatory variables and further refined the models through backwards, stepwise selection. We subsequently validated the models using established methods [66].

#### NC Zebra and Springbok

Similar to the GEE models built for captured zebra, we used each parasite type and FGM as response variables in their own models (Tables E and F in S1 File) to determine how seasonal factors, host immunity, hormone concentrations, and coinfections affected the intensity and prevalence of each pathogen, or how they affected stress hormone concentrations. In addition, because there were several missing variables for many NC zebra and springbok samples (see discussion above), we built a series of nested models for each response parameter. This allowed us to take advantage of the largest possible dataset for each iteration. We began with models of the response variable in question explained solely by simple host and environmental factors (rainfall, age, sex) and FGM (for parasite models) or by those factors and the GI parasite counts (for FGM models). This allowed us to use a sample size of 302 for NC zebra and greater than 260 for springbok (Tables E and F in S1 File). We then added the FPM and FEM reproductive hormones to each model (*N* = 163 complete cases for NC zebra; *N* = 113–114 for springbok). Finally, we built males-only models to account for the effects of FTM on parasite counts and FGM concentrations (*N* = 103 complete cases for NC zebra; *N* = 77 for springbok) (Tables E and F in S1 File).

We developed generalized linear models (GLM) using R v2.15.2 and the 'MASS' package [68] to examine the correlations between parasite types (Strongyles, *Strongyloides* and *Coccidia* for springbok; Strongyles for zebra), seasonal factors and hormone metabolite concentrations. We used negative binomial (NB) GLMs with a log link function for NC zebra GIP models and springbok GIP and GIC models to deal with large amounts of overdispersion in these counts variables [66] (Tables E and F in S1 File). We used a gamma distribution with an inverse link function for FGM GLMs for both NC zebra and springbok (Tables E and F in S1 File). For *Strongyloides* parasites (GIS) models, zero inflated negative binomial (ZINB) mixture models were used to deal with the large numbers of zero GIS counts (80 samples out of 273, 29.3%) and the large overdispersion present in these data [66] (Table F in S1 File); We used the 'pscl' package in R to develop these models [69]. We verified that ZINB models were more useful for modeling the GIS data than were regular negative binomial GLMs by applying Vuong tests to compare both types of models; a high positive Vuong statistic favors the ZINB over the NB GLM [70].

We developed each GLM or ZINB model by using a backwards, stepwise refinement method based on comparing the AIC values between maximal models and models with variables removed. After stepwise selection of the main terms, we added interaction terms between the remaining explanatory variables and further refined the models through backwards, stepwise selection. We validated the models using previously established methods [66]. For the final, best-fit ZINB models, we determined the fitted values for the logistic regression model components and determined the odds ratios of observing excess false zeros in conjunction with each predictive parameter [71].

## Results

### Seasonality of Pathogen Prevalence, Immune Measures, and Hormone Concentrations

#### Captured Zebras

**Fig 1:**
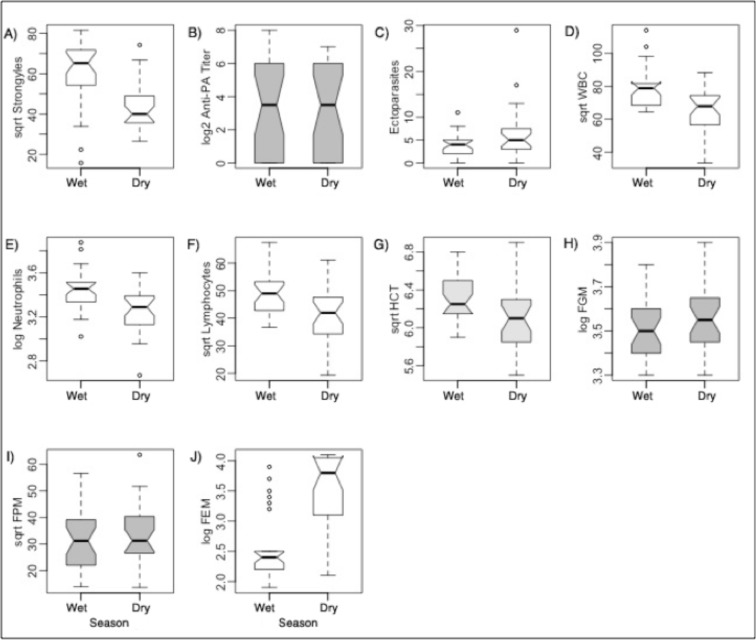
Pairwise comparisons of pathogens, immune factors, and hormones between rain groups for the same captured zebra individuals resampled twice. Center lines in boxplots represent medians, with notches extending to ±1.58 IQR/$\sqrt{N}$ where IQR is the interquartile range and *N* is the sample size. Notches that do not overlap provide strong evidence that the medians differ (Chambers et al. 1983) (not shown for log2PA because notches grossly overlapped the ends of the boxes). The box hinges represent nearly the first and third quartiles, with whiskers showing the largest and smallest observations 1.5 times the box size from the nearest hinge. Points outside this range are shown as open circles. Boxplots in white are for variables that are significantly different from each other by *t* tests or Wilcoxon rank sum tests, whereas gray boxplots are not significantly different. sqrtHCT is light gray because the rain group for this variable was nearly significant (*p* = 0.080).

For both unique animals and paired recaptures, we found statistically significantly higher GI strongyle counts, total white blood cell counts, and neutrophil counts in the wet season (Tables G and H in S1 File; Fig. 1). Zebra experienced significantly higher estrogen concentrations in the dry season, indicating that the majority of mares were in mid-gestation in the middle of the hot dry season (Fig. 1).

We found higher lymphocyte counts in the wet season, but only for paired individuals (Tables G and H in S1 File; Fig. 1); this indicates that controlling for individual variation allowed these subtler, yet present seasonal differences to be revealed. HCT was significantly and nearly significantly higher in the wet season for unique and paired animals, respectively. Stress hormone concentrations were also significantly higher in drier seasons compared to in the wetter ones for paired animals, though only prior to the Holm's *p* correction; this corroborates possibly higher stress in the dry season observed for unique animals.

Thus, overall, GI strongyle infection intensities, total white blood cell counts, lymphocyte and neutrophil cell counts, and HCT were higher in the wet season for these animals, whereas stress hormone concentrations were higher in the dry season while most mares were in mid-gestation.

#### Non-Captured Zebras

Similar to the results for comparisons in captured zebra, we found that GI strongyle counts were significantly higher in wetter seasons that in drier ones. The highest overall GIP infection intensities were males and yearlings (Table I in S1 File).

**Fig 2:**
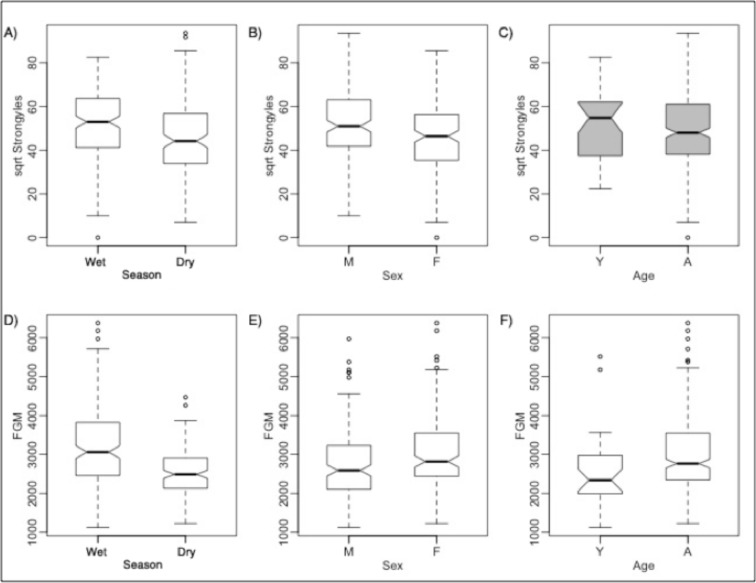
Comparisons of Strongyle counts and stress hormone metabolites (FGM) between rain groups, sexes, and ages for non-captured zebra. For clarity, comparisons between interaction effects are not shown. Boxplot statistics are interpreted as in Fig. 1. Boxplots in white are for variables that are significantly different from each other by Tukey's *t* tests or Wilcoxon rank sum tests, whereas gray boxplots are not significantly different. Though non-transformed parasite counts (GIP) were compared with Wilcoxon rank sum tests, GIP is square root transformed here (as close to normality as possible) for clarity.

**Fig 3:**
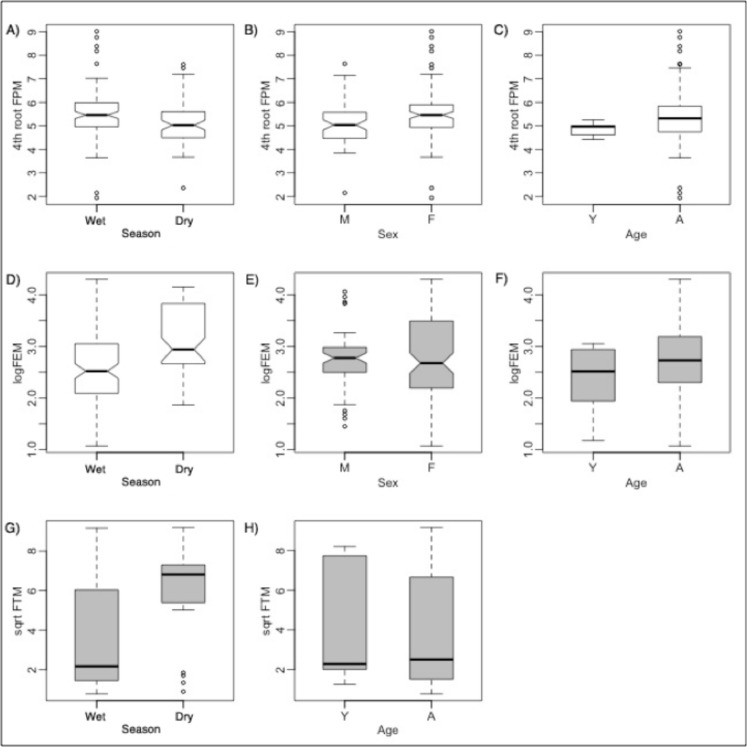
Comparisons of reproductive hormones between rain groups, sexes, and ages for non-captured zebra. For clarity, comparisons between interaction effects are not shown. Boxplot statistics are interpreted as in Fig. 1. Boxplots in white are for variables that are significantly different from each other by Tukey's *t* tests or Wilcoxon rank sum tests, whereas gray boxplots are not significantly different. Though non-transformed FPM and FEM data were compared with Wilcoxon rank sum tests, they are fourth root and log transformed here (as close to normality as possible) for clarity.

Estrogen concentrations were significantly higher during the dry seasons (Fig. 2), and progesterone was significantly higher in wetter seasons (Fig. 3). Testosterone concentrations were not significantly different between seasons. Females had significantly higher stress hormone concentrations than did males, overall and for two age group comparisons (Table I in S1 File; Fig. 2). Adults in general had higher stress hormone levels.

Thus, overall, GI strongyle infection intensities were highest in the wet season and in yearling males, with males overall carrying more parasites than did females. Most mares were in mid-gestation in the dry season, and adult females had the highest stress hormone concentrations.

#### Springbok

**Fig 4:**
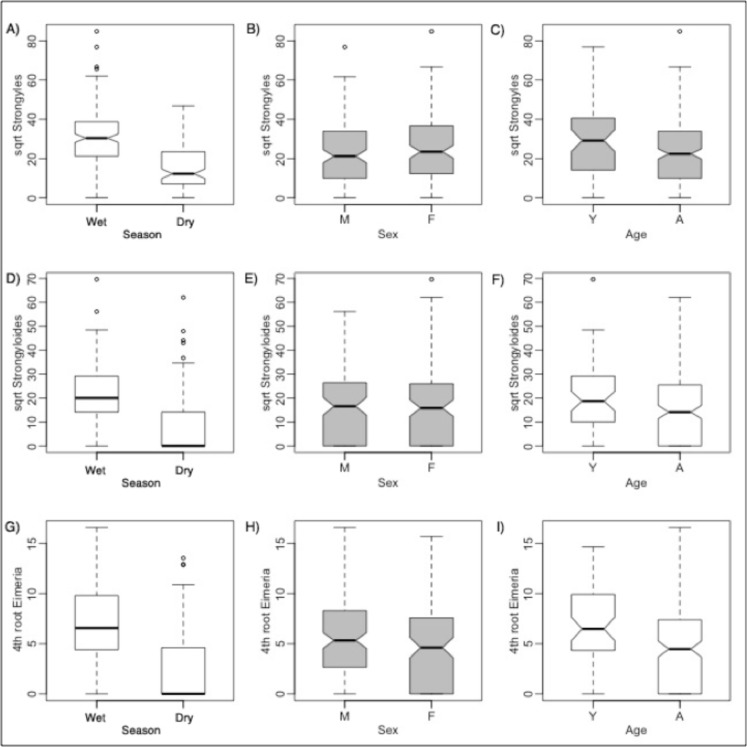
Comparisons of parasites between rain groups, sexes, and ages for springbok. For clarity, comparisons between interaction effects are not shown. Boxplot statistics are interpreted as in Fig. 1. Boxplots in white are for variables that are significantly different from each other by Tukey's *t* tests or Wilcoxon rank sum tests, whereas gray boxplots are not significantly different. Though non-transformed parasite counts for all parasites were compared with Wilcoxon rank sum tests, they are fourth root and square root transformed here (as close to normality as possible) for clarity.

Similar to the results for comparisons in captured and NC zebra, we found that GI strongyle counts were significantly higher in wetter seasons. *Strongyloides* and *Eimeria* counts were also both significantly higher in springbok in the wet season compared to in the dry (Fig. 4). Yearlings harbored significantly more *Strongyloides* and *Eimeria* parasites than did adults. Male yearlings had significantly higher strongyle counts than did male and female adults, and possibly more than did female yearlings.

**Fig 5:**
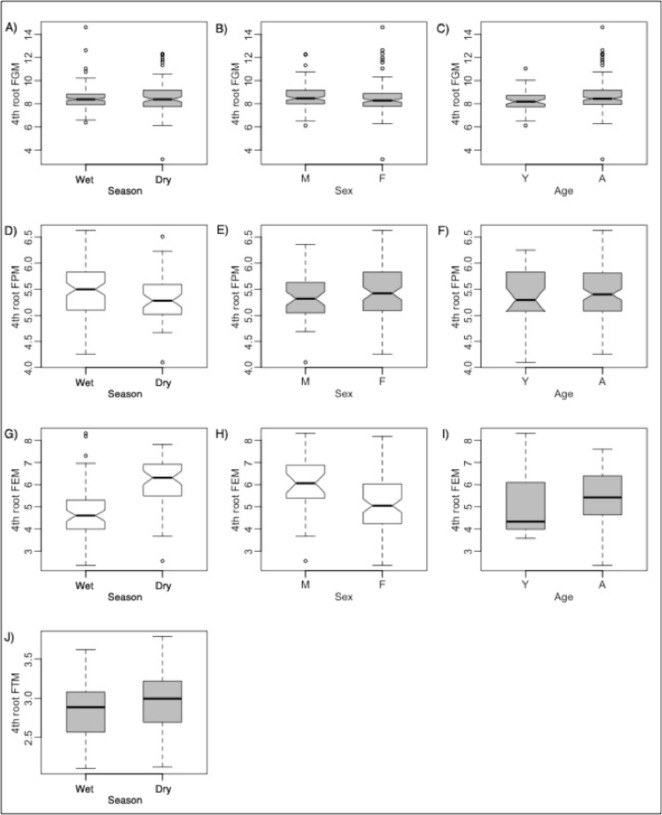
Comparisons of hormones between rain groups, sexes, and ages for springbok. For clarity, comparisons between interaction effects are not shown. Boxplot statistics are interpreted as in Fig. 1. Boxplots in white are for variables that are significantly different from each other by Tukey's *t* tests or Wilcoxon rank sum tests, whereas gray boxplots are not significantly different. Though non-transformed FGM data were compared with Wilcoxon rank sum tests, they are fourth-root transformed here (to help meet assumptions of normality) for clarity.

**Fig 6:**
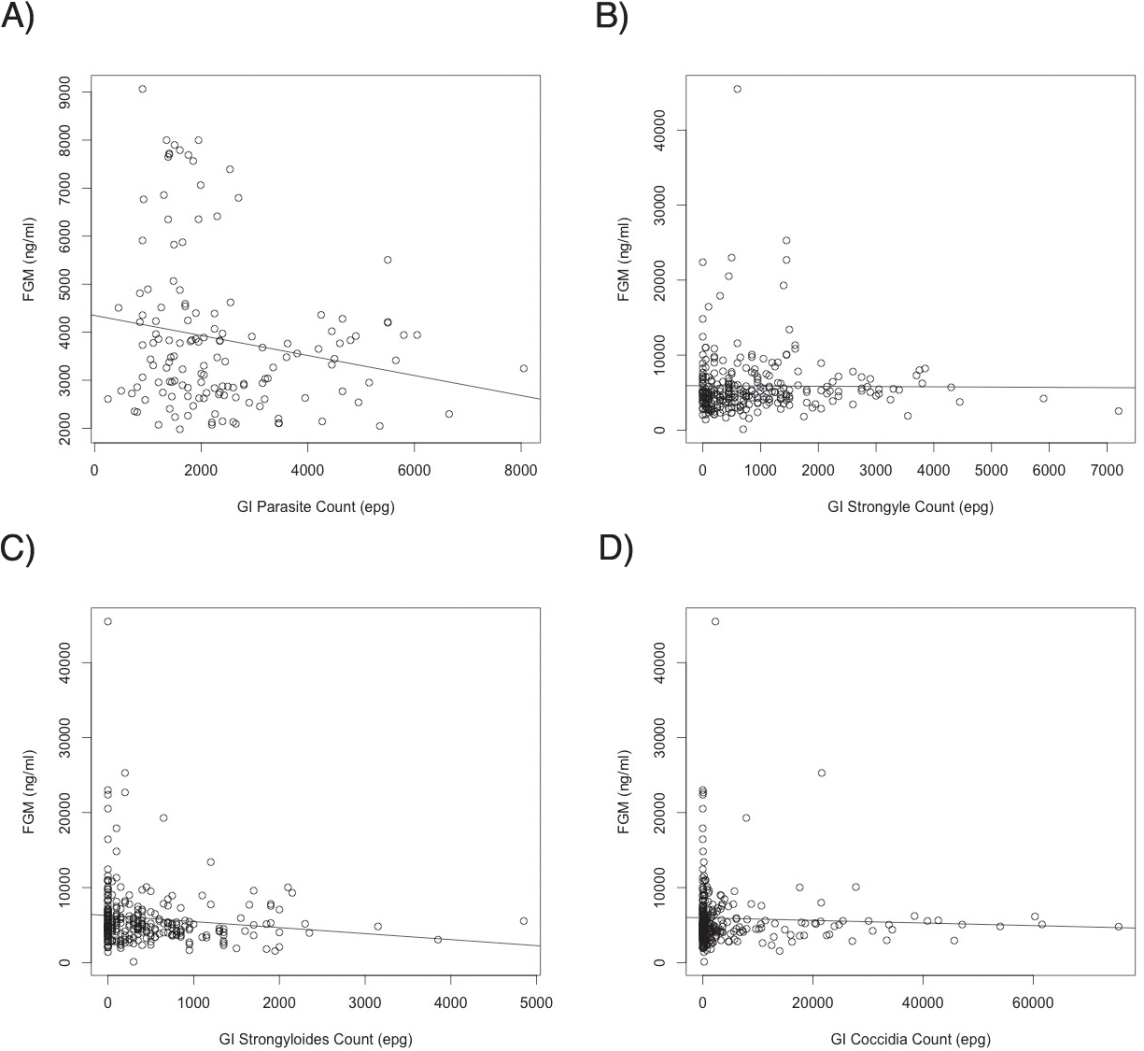
Tolerance curves indicating the relationship between a measure of host health and pathogen load. FGM (a measure of host chronic stress responses) is used here as a proxy of host health, and is measured against the range of various GI parasite types found in these hosts. A) Zebra and strongyle parasites; B) Springbok and strongyle parasites; C) Springbok and *Strongyloides* parasites; D) Springbok and coccidian parasites. With a lack of host tolerance to these parasites, one would expect to see an increase in FGM (decrease in host health) with increased parasite infection intensities (as indicated by increasing epg—eggs per gram of feces—counts) [101] Instead, these hosts experience either no increase in FGM, or a decrease in FGM with increased parasite infection intensities in all instances.

Estrogen concentrations were significantly higher during the dry seasons (Table J in S1 File; Figs. 5 and 6). Similar to the trends measured in NC zebra, springbok progesterone concentrations were significantly higher in the wet season (Fig. 5).

Thus, overall, GI parasite infection intensities of all three kinds of parasites were higher in the wet season, with male yearlings being the highest parasite aggregators. The majority of adult ewes were in mid-gestation during the dry season, similar to zebra mares.

### Interrelationships Between Pathogens, Physiology, and Season

#### Captured Zebras

*Pathogen Interactions*: Higher cumulative rainfall two months prior to sampling (hereafter simply "rainfall") predicted higher GI parasite loads (GIP), as expected from rain group analyses (Fig. 1). Mares in full milk were more likely to harbor more strongyles than were those lactating water or not lactating at all. As FEM increased, PA titer prevalence decreased. Strongyle counts also negatively predicted anti-PA titers, as we have previously demonstrated [18] (Table 1). Thus, overall, GI strongyles were highest in the wet season and more intense infections were associated with lactation, and estrogen and strongyle infections were negatively associated with host ability to mount an anti-anthrax immune response.

**Table 1:** Maximum likelihood estimates for the best fit generalized estimating equation pathogen models for captured zebras.

Response	Coefficients	Estimate ± SE	Wald statistic	***p*-value**	Δ QIC from full model
GIPsqrt	**Intercept**	35.5 ± 2.36	227	0.000***	454
	**Rain2**	0.09 ± 0.01	52.3	0.000***	
	**Neut**	-0.01 ± 0.01	5.47	0.019*	
	**Lymph**	0.01 ± 0.01	11.1	0.001***	
	**log2PA**	-0.72 ± 0.34	4.47	0.035*	
	**Lact**	3.26 ± 0.94	12.0	0.001***	
PA	**Intercept**	-2.55 ± 0.71	12.5	0.001***	11.1
	Rain2	-6.92e-05 ± 6.13e-04	0.01	0.910	
	**Lymph**	1.36e-04 ± 6.15e-05	4.88	0.027*	
	**logFGM**	0.88 ± 0.20	20.5	0.000***	
	**FEM**	-2.55e-05 ± 8.95e-06	8.09	0.005**	
	GIP	-4.08e-05 ± 3.33e-05	1.50	0.221	
	Rain2*FEM	2.77e-07 ± 9.95e-08	7.77	0.005**	
Ectosqrt	**Intercept**	1.55 ± 0.22	51.3	0.000***	11
	Rain2	3.78e-04 ± 1.02e-03	0.14	0.711	
	FEM	4.10e-05 ± 2.12e-05	3.75	0.053.	
	**Preg**	0.56 ± 0.20	7.72	0.006**	
	**Lact**	0.21 ± 0.08	7.04	0.008**	
	Rain2*Preg	-2.47e-03 ± 1.05e-03	5.53	0.019*	
	Rain2*FEM	-3.96e-07 ± 2.12e-07	3.48	0.062.	

Significant coefficients (*p*< 0.05) are in bold.

*p*≤0.1

*p≤0.05

**p≤0.01

***p≤0.001

*Immune Interactions*: Higher rainfall significantly predicted higher neutrophil counts (Table 2), corroborating similar trends we observed with our group comparisons (Fig. 1). Lactating mares were more likely to have higher neutrophil counts, whereas those with higher estrogen levels were negatively associated with neutrophil counts (Table 2). Contrary to what we observed in our group comparisons, increased rainfall in the GEE model predicted that hosts would carry fewer lymphocytes (Table 2). Neutrophils and strongyles positively predicted lymphocyte counts, similar to the relationships we observed in our neutrophil and GIP models. Pregnant mares had significantly fewer lymphocytes. Thus, overall, zebra in the wet season had higher neutrophil counts but lower lymphocyte counts, estrogen and pregnancy were associated with lower specific immune cell counts, and animals with higher strongyle infection intensities had higher lymphocyte counts.

**Table 2:** Maximum likelihood estimates for the best fit generalized estimating equation immunity and hormone models for captured zebras.

Response	Coefficients	Estimate ± SE	Wald statistic	***p*-value**	Δ QIC from full model
logNeut	**Intercept**	2.70 ± 0.14	361	0.000***	5.9
	**Rain2**	6.29e-04 ± 2.00e-04	9.94	0.002**	
	**Lymph**	1.51e-04 ± 1.80e-05	70.7	0.000***	
	**HCT**	7.14e-03 ± 3.25e-03	4.84	0.028*	
	**FPM**	6.56e-05 ± 1.45e-05	20.5	0.000***	
	**FEM**	-6.95e-06 ± 3.43e-06	4.11	0.043*	
	**GIP**	-2.65e-05 ± 8.26e-06	10.3	0.001***	
	**Ecto**	-5.78e-03 ± 2.64e-03	4.78	0.029*	
	**Lact**	0.03 ± 0.01	6.91	0.009**	
Lymphsqrt	**Intercept**	-50.0 ± 6.47	59.9	0.000***	5.8
	**Rain2**	-0.01 ± 5.81e-03	4.88	0.027*	
	**logNeut**	29.3 ± 1.87	246	0.000***	
	**GIPsqrt**	0.12 ± 0.04	9.84	0.002**	
	**Age**	-2.85e-03 ± 5.72e-04	24.9	0.000***	
	**Preg**	-1.58 ± 0.49	10.4	0.001***	
logFGM	**Intercept**	3.43 ± 0.03	12259	0.000***	10.8
	**Rain2**	-3.60e-04 ± 1.67e-04	4.66	0.031*	
	FPM	2.72e-05 ± 1.79e-05	2.31	**0.129**	
	**FEM**	1.08e-05 ± 2.70e-06	16.0	0.000***	
	**log2PA**	0.01 ± 4.10e-03	8.59	0.003**	
	Rain2*FPM	2.57e-07 ± 1.23e-07	1.23e-07	0.037*	
Agesqrt	**Intercept**	52.3 ± 1.60	1068	0.000***	378
	**Lymph**	-2.12e-03 ± 5.65e-04	14.15	0.000***	
	**FPM**	6.05e-04 ± 2.02e-04	8.97	0.003**	
	log2PA	-0.18 ± 0.21	0.76	0.382	
	GIP	-1.54–04 ± 2.01e-04	0.59	0.443	
	Lymph*log2PA	2.94e-04 ± 1.17e-04	6.30	0.013*	
	log2PA*GIP	-9.03e-05 ± 4.20e-05	4.62	0.032*	

Significant coefficients (*p*≤ 0.05) are in bold.

*p*≤0.1

*p≤0.05

**p≤0.01

***p≤0.001

*Hormone Interactions*: Rainfall negatively predicted stress hormone levels in our GEE model (Table 2). Both higher rainfall and higher progesterone levels together, however, predicted higher FGM. These opposite effects between rainfall and its interaction with FPM may reflect gestational timing factors. In accordance with this idea, increasing FEM positively predicted FGM levels. Thus, overall, the wet season was predominantly associated with less stress in zebra hosts, though zebra in the periparturient period in the wet season experienced higher stress, as did those with the highest estrogen levels.

#### NC Zebras

*Pathogen Interactions*: In accordance with all other zebra strongyle models and comparisons, more rainfall predicted higher GI parasite loads in all GIP models (Table 3). However, higher rainfall in conjunction with higher stress hormone concentrations predicted less intense strongyle infections in the GIP-hormones model. This relationship was perhaps driven primarily by the stress effects; higher stress levels also significantly, negatively predicted strongyle counts in the simplest GIP-FGM model, corroborating the interaction effects in the more complex GIP-hormones model. This highly significant, negative relationship between FGM and strongyle count also held for the GIP-males only model (Table 3). Males were more likely to have higher strongyle counts in the simplest GIP-FGM model, similar to what we observed in our group comparisons (Fig. 2). Thus, overall, these models indicate that strongyles in zebras were predominantly positively associated with rainfall and males, and were negatively associated with stress hormone concentrations and females.

**Table 3:** Maximum likelihood estimates for the best fit generalized linear models for non-captured zebras.

Response	Coefficients	Estimate ± SE	***z* Value**	Pr(**>|*z*|**)	***N***	ΔAIC
GIP	**Intercept**	8.12 ± 0.12	69.0	0.000***	302	2.00
	**Rain2**	2.19e-03 ± 3.84e-04	5.71	0.000***		
	**Sex**	-0.16 ± 0.07	-2.17	0.030*		
	**FGM**	-1.57e-04 ± 4.31e-05	-3.63	0.000***		
GIP	**Intercept**	7.70 ± 2.31e-03	17.5	0.000***	163	13.7
	Rain2	2.79e-03 ± 2.31e-03	1.21	0.228		
	FGM	2.35e-04 ± 1.21e-04	1.94	0.052.		
	**logFEM**	-0.35 ± 0.12	-2.80	0.005**		
	rain2*FGM	-1.69e-06 ± 5.64e-07	-3.00	0.003**		
	rain2*logFEM	1.80e-03 ± 6.43e-04	2.80	0.005**		
GIP (males)	**Intercept**	8.15 ± 0.17	48.6	0.000***	103	2.30
	**Rain2**	3.34e-03 ± 5.05e-04	6.612	0.000***		
	**FGM**	-2.55e-04 ± 6.26e-05	-4.07	0.000***		
FGM	**Intercept**	5.20e-04 ± 5.42e-05	9.59	0.000***	302	8.30
	**Rain2**	-3.25e-07 ± 9.06e-08	-3.59	0.000***		
	**AgeC**	-7.77e-05 ± 2.63e-05	-2.95	0.003**		
	**Sex**	-2.27e-04 ± 7.55e-05	-3.01	0.003**		
	**GIP**	2.29e-08 ± 6.82e-09	3.36	0.001***		
	AgeC*Sex	1.08e-04 ± 3.89e-05	2.78	0.006**		
	Rain2*GIP	-6.74e-11 ± 3.43e-11	-1.96	0.051.		
FGM	**Intercept**	4.52e-04 ± 5.21e-05	8.68	0.000***	163	3.30
	**Rain2**	-4.21e-07 ± 5.79e-08	-7.28	0.000***		
	Sex	2.22e-05 ± 1.17e-05	1.91	0.058.		
	**GIP**	4.00e-08 ± 1.92e-08	2.08	0.039*		
	**FPM4**	-1.60e-05 ± 5.88e-06	-2.72	0.007**		
	logFEM	-6.04e-06 ±1.69e-05	-0.36	0.721		
	GIP*logFEM	-1.24e-08 ± 6.98e-09	-1.78	0.077.		
FGM (males)	**Intercept**	3.64e-04 ± 2.99e-05	12.2	0.000***	103	6.50
	**Rain2**	-2.52e-07 ± 1.20e-07	-2.10	0.038*		
	**GIP**	4.98e-08 ± 1.15e-08	4.34	0.000***		
	**FTM**	-1.27e-06 ± 3.15e-07	-4.04	0.000***		
	Rain2*GIP	-1.46e-10 ± 4.97e-11	-2.94	0.004**		

Significant coefficients (*p*≤ 0.05) are in bold.

As there were several missing variables for many NC zebra, we built a series of nested models for each response parameter, allowing us to take advantage of the largest possible dataset for each iteration (see the differing *N* for each model). The first model in each set includes only host and environmental factors and either FGM (parasite models) or GI parasite counts (FGM models). The second model in each set also includes FPM and FEM hormones. The final model in each set includes only males to examine FTM effects. Abbreviations are explained in Table C in S1 File.

*p*≤0.1

*p≤0.05

**p≤0.01

***p≤0.001

*Hormone Interactions*: More rainfall predicted lower stress hormone concentrations in all models, in concordance with the patterns seen in captured zebra (Table 2). In all three FGM models, higher strongyle counts predicted higher stress. However, higher stress levels predicted lower strongyle counts in GIP models; this negative correlation was of greater magnitude than was the positive correlation between strongyles and stress in the FGM models. (Table 3). In addition, rainfall-GIP interaction terms were nearly and highly significant in negatively predicting stress hormone levels in both the FGM-GIP and the FGM-males only models, respectively. Older females significantly predicted higher stress hormone concentrations in the FGM-GIP model, and males with higher testosterone levels were associated with lower FGM in the FGM-males only model. These results, taken together with the fact that females had significantly higher FGM in group comparisons (Fig. 2), suggest that female zebras likely do experience higher stress hormone levels than do males. Thus, overall, stress hormone concentrations were higher in the dry season (as they were in the captured zebra group), and stress hormones were typically negatively associated with GI strongyle infection intensity and maleness.

#### Springbok

*Pathogen Interactions*: In all GIP models, higher rainfall predicted more strongyles (Table 4). *Strongyloides* counts were inversely associated with rainfall in the final GIS-FGM model; however, increased rainfall also predicted fewer false zeros in *Strongyloides* counts, indicating a complex relationship between these parasites and rainfall (Table 4). A simple linear model of rainfall on transformed GIS was highly significant and indicated a positive relationship between rainfall and *Strongyloides* infection intensities (*N* = 266, *F*_1,264_ = 45.9, *R* ^2^ = 0.15, *p* = 0.000, coeffiecient = 0.06). *Eimeria* counts were associated with high rainfall and younger animals. Thus, overall, macroparasite infection intensities in springbok were highest in the wet season.

**Table 4:** Maximum likelihood estimates for the best fit generalized linear models for springbok helminths.

Response	Coefficients	Estimate ± SE	***z* Value**	Pr(**>|*z*|**)	***N***	ΔAIC
GIP	**Intercept**	4.50 ± 0.22	20.6	0.000***	267	18.8
	**Rain2**	0.01 ± 1.16e-03	8.93	0.000***		
	**GISsqrt**	0.08 ± 0.012	6.73	0.000***		
	FGM	4.46e-05 ± 2.30e-05	1.94	0.052.		
	Rain2*GISsqrt	-3.67e-04 ± 7.87e-05	-4.67	0.000***		
GIP	**Intercept**	4.74 ± 0.24	19.4	0.000***	114	10.9
	**Rain2**	0.01 ± 0.01	5.75	0.000***		
	**GISsqrt**	0.09 ± 0.02	3.74	0.000***		
	Rain2*GISsqrt	-4.49e-04 ± 1.43e-04	-3.14	0.002**		
GIP (males)	**Intercept**	4.75 ± 0.26	18.3	0.000***	77	44.2
	**Rain2**	8.49e-03 ± 1.89e-03	4.50	0.000***		
	**GISsqrt**	0.10 ± 0.03	4.09	0.000***		
	Rain2*GISsqrt	-3.87e-04 ± 1.44e-04	-2.69	0.007**		
GIS Counts	**Intercept**	6.64 ± 0.21	32.4	0.000***	267	41.7
	**Rain2**	-3.00e-03 ± 9.78e-04	-3.06	0.002**		
	**GIC4**	0.07 ± 0.02	3.50	0.001***		
	**FGM**	-1.63e-05 ± 1.13e-06	-40.1	0.000***		
GIS Zeros	**Intercept**	2.80 ± 0.56	5.01	0.000***		
	**Rain2**	-9.30e-03 ± 3.10e-03	-3.00	0.003**		
	**GIPsqrt**	-0.08 ± 0.02	-3.6	0.000***		
	**GIC4**	-0.30 ± 0.09	-3.19	0.001***		
GIS Counts	**Intercept**	6.65 ± 0.16	43.0	0.000***	114	4.33
	**FEM**	-2.13e-04 ± 8.12e-05	-2.62	0.009**		
GIS Zeros	**Intercept**	3.16 ± 1.09	2.89	0.004**		
	**GIPsqrt**	-0.15 ± 0.04	-3.66	0.000***		
	**GIC4**	-0.61 ± 0.17	-3.58	0.000***		
	**FEM**	1.05e-03 ± 4.34e-04	2.42	0.016*		
GIS (males) Zeros	**Intercept**	-2.97 ± 0.73	-4.05	0.000***	77	2.30
	**GIPsqrt**	0.11 ± 0.03	3.53	0.000***		
	**GIC4**	0.29 ± 0.10	2.90	0.004**		

Significant coefficients (*p*≤ 0.05) are in bold.

The series of nested models (with different *N*) follow the same patterns listed in the caption for Table 3.

*p*≤0.1

*p≤0.05

**p≤0.01

***p≤0.001

*Hormone Interactions*: Higher rainfall significantly, positively predicted higher stress hormones in the FGM-parasites model (Table 5). Higher stress hormones nearly significantly (*p* = 0.052) predicted higher strongyle counts in the GIP-FGM model; however, strongyle loads significantly, negatively predicted FGM in FGM models, similar to the inverse relationship between GIP and FGM in most NC zebra GIP models (Table 3). Increased stress hormone concentrations significantly predicted fewer *Strongyloides* in the GIS-FGM model (Table 4), and higher rainfall in conjunction with higher GIS significantly, negatively predicted FGM (Table 5), corroborating trends observed in GIS models. *Eimeria* counts alone did not significantly influence FGM in any models, and stress and other hormones did not play a role in predicting *Eimeria* counts in *Eimeria* models (Table 5). While higher testosterone concentrations in males predicted lower stress, higher *Eimeria* counts in conjunction with higher testosterone levels predicted higher stress hormone concentrations. Finally, females with high estrogen levels significantly predicted higher stress hormone concentrations in the FGM-hormones model. Thus, overall, higher stress hormone concentrations were associated with the wet season and estrogen, and were negatively associated with strongyles and *Strongyloides*. Stress hormones were not associated with *Eimeria* infections except in males with both high testosterone levels and *Eimeria* infections.

**Table 5:** Maximum likelihood estimates for the best fit generalized linear models for springbok *Eimeria* and stress hormones.

Response	Coefficients	Estimate ± SE	***z* Value**	Pr(**>|*z*|**)	***N***	ΔAIC
GIC	**Intercept**	5.77 ± 0.68	8.44	0.000***	267	12.7
	**Rain2**	0.019 ± 1.924e-03	10.0	0.000***		
	**AgeC**	-0.74 ± 0.33	-2.25	0.025*		
	**GISsqrt**	0.12 ± 0.02	6.08	0.000***		
	Rain2*GISsqrt	-5.10e-04 ± 1.30e-04	-3.93	0.000***		
GIC	**Intercept**	4.50 ± 0.34	13.4	0.000***	114	8.90
	**Rain2**	0.014 ± 2.07e-03	6.54	0.000***		
	**GISsqrt**	0.05 ± 0.02	2.64	0.008**		
GIC (males)	**Intercept**	3.17 ± 0.38	8.32	0.000***	77	7.10
	**Rain2**	0.02 ± 2.77e-03	8.37	0.000***		
	**GISsqrt**	0.19 ± 0.04	5.13	0.000***		
	Rain2*GISsqrt	-7.66e-04 ± 2.11e-04	-3.63	0.000***		
FGM	**Intercept**	2.30e-04 ± 3.35e-05	6.863	0.000***	263	27.9
	**Rain2**	2.91e-07 ± 9.60e-08	3.032	0.003**		
	**AgeC**	-3.58e-05 ± 1.63e-05	-2.205	0.028*		
	**GIPsqrt**	-1.51e-06 ± 4.41e-07	-3.420	0.001***		
	**GISsqrt**	3.23e-06 ± 9.54e-07	3.390	0.001***		
	Rain2*GISsqrt	-1.40e-08 ± 5.85e-09	-2.393	0.017*		
FGM	**Intercept**	2.62e-04 ± 3.87e-05	6.78	0.000***	113	14.1
	Sex	-5.61e-05 ± 4.16e-05	-1.35	0.181		
	**FEM**	-6.45e-08 ± 1.63e-08	-3.96	0.000***		
	Sex*FEM	4.94e-08 ± 1.90e-08	2.61	0.011*		
FGM (males)	**Intercept**	2.42e-04 ± 3.40e-05	7.11	0.000***	77	5.70
	GIC4	-3.43e-06 ± 5.07e-06	-0.68	0.501		
	**FTM**	-1.08e-06 ± 2.98e-07	-3.64	0.001***		
	GIC4*FTM	9.65e-08 ± 5.44e-08	1.78	0.080.		

Significant coefficients (*p*≤ 0.05) are in bold.

The series of nested models (with different *N*) follow the same patterns listed in the caption for Table 3.

*p*≤0.1

*p≤0.05

**p≤0.01

***p≤0.001

## Discussion

This study evaluated the seasonality of stress responses in conjunction with environmental, reproductive, parasitic, and immunologic factors. We aimed to determine how the strong seasonality of environmentally transmitted parasites such as helminths and coccidian parasites correlated with changes in host reproduction, immune function, and measures of chronic stress, as well as with the occurrence of coinfections. As we have previously found that wet season-driven GI strongyles are likely immunomodulatory in zebra hosts and therefore may influence susceptibility to anthrax in Etosha National Park [18], we wanted to determine if these pathogens also influence host susceptibility by invoking stress responses and stress-related immunosuppression. In addition, such correlations would provide evidence that these GI parasites likely affect host fecundity and survival through mechanisms other than direct pathology and resource use. Instead, we found that, while stress responses do exhibit seasonality in zebra, stress hormone levels largely peak in the dry season when parasite infection intensities are lowest. Stress responses in springbok were more complex, with stress alone positively related to rainfall in some models, but significantly, negatively related to rainfall in conjunction with helminth infection intensities, and unrelated to coccidian infections. Rather than by parasite prevalence or infection intensity, peak stress hormone levels are most likely driven by dry season environmental challenges and host reproductive status in both species examined.

### Seasonal Patterns

As expected, we found strong seasonality in nearly all parameters examined. Previous studies in this system have found that GI parasite infection intensities in zebra and springbok peak in the wet season, and that prevalence of strongyles, *Strongyloides*, and *Eimeria* in springbok reaches nearly 90% during times of highest rainfall [13]. While the seasonality of GI macroparasites in ENP is largely determined by environmental conditions [45,72,73], transmission rates can change seasonally due to changes in host behaviors and the birth of large numbers of immune naïve hosts, and host susceptibility can change due to physiological factors such as reproductive hormonal fluxes and changing nutrition [74,75]. Thus, our examination of the seasonality of these other factors was also important for determining what may influence parasite infections, and how parasite infections may interact temporally with physiological changes.

Though technically nonseasonal breeders [23], both zebra and springbok in ENP have been observed to breed on a largely seasonal basis, with birth peaks in the wet season [11,13]. Estrogen in pregnant zebras peaks in mid-gestation before declining sharply in the last trimester [21,51]. Estrogen also reaches a sustained peak in mid-pregnancy in springbok, with progesterone peaking just prior to parturition [22,76]. Our findings that FEM levels were significantly, negatively associated with rainfall for both species therefore indicates that the majority of the zebra and springbok examined were in mid-gestation in the hot dry season (late September-early November), whereas the significantly increased FPM in the wet season indicates that the majority of females of both species were giving birth and/or coming into estrus at this time. Thus, this study provides definitive hormonal evidence of strong reproductive seasonality for both of these species in this system. Interestingly, both male zebra and springbok in the dry season had significantly higher FEM than did females in the wet season, indicating, unlike in other similar studies [51], that there is likely an environmental, seasonal component to reproductive hormone secretion as well.

We also found seasonal patterns in WBC counts, HCT, lymphocytes, and neutrophils, in corroboration with the seasonality of several other immune factors previously measured in these zebra [18]. Higher HCT in the wet season likely reflects a higher nutritional state, especially in light of the fact that dehydration often results in an elevated HCT [77]. Higher WBC in the wet season may also reflect this higher plane of nutrition, as higher WBCs in general often represent a higher investment in immunity [78].

Despite individuals experiencing significantly more intense macroparasite infections in the wet season, higher stress hormones were overall correlated with decreased rainfall, particularly for zebras. Some of this seasonality was likely driven by environmental factors such as decreased nutrition and water availability in the dry season. Stress hormones may have been more seasonally linked in zebra than in springbok due to the fact that zebra are much more water-dependent than the more arid-adapted springbok [79,80]; zebras may be able to persist at their relatively high population level in ENP due, in part, to the fact that man-made water holes provide accessible drinking water year-round [33]. Elevations of GCs in the face of altered nutrition could be adaptive to help mobilize energy stores, rather than indications of distress: there is evidence that predictable aversive conditions such as seasonal changes may cause animals to preemptively raise GC levels [2]. However, the interaction between FGM and other factors during the dry season indicates that animals also likely do experience adverse chronic stress responses at this time, more so than they do during the wet season. Our more complex models examining the interrelationships between variables helped to shed light on the potential sources of these patterns (discussed below).

### Stress and Reproduction

Stress hormone levels were not only higher during the same season most females were in mid-gestation, but were also significantly correlated with reproductive hormone concentrations. Increased FEM significantly predicted higher FGM for zebra (Table 2), and increased FEM predicted higher FGM for female springbok (Table 5). Thus, it appears that pregnancy and its attendant hormone increases correlate with a significant stress response in these animals. The lack of strong correlations in this study between researcher-determined pregnancy status and FGM are likely due to the subjective nature of pregnancy assessment by visual inspection and light palpation. While there are many studies illustrating the effects of stress on reproductive hormone concentrations and reproductive success, this is one of the first studies (also see [81]) examining how pregnancy itself may correlate with chronic stress in wildlife. Progesterone in zebras peaks in the last 50 days prior to foaling, and declines sharply in the short periparturient period [51,53].

In models examining only female zebra (captured animals), FPM was positively associated with FGM, and high rainfall interacting with high FPM significantly predicted increased stress hormone levels (Table 2); this likely reflects females in the periparturient period and just prior to estrus, and the distinct possibility that these reproductive efforts cause stress in mares. Progesterone in springbok was not related to stress, perhaps because these animals tend to give birth earlier in the wet season and mate in the early dry season, while samples examined in this study were predominantly taken from the mid wet season. In accordance with these findings, female zebras overall had higher stress hormone levels than did males. Other wildlife studies have found similar sex-stress patterns and have suggested that, in addition to the stress of reproduction, higher GC secretion in females may be adaptive to promote longer-term survival and the energy demands of reproduction [26].

### Reproduction, Pathogens, and Immunity

Gestation in this study correlated with significantly higher stress hormone concentrations in female animals. While such chronic stress often leads to immunosuppression, and while immunosuppression can lead to increased disease susceptibility, we did not find evidence for this kind of cascade in our study. Instead, we found that reproductive hormones themselves influenced immunity more directly, and that reproductive hormones and sex were important factors in predicting pathogen infection intensity. Non-pregnant zebras had higher lymphocyte counts, and lower FEM was significantly correlated with higher neutrophil counts. Breeding and immunity are both expensive and somewhat incompatible, as elevations of one often compromise the other [82]. However, despite the fact that lactation and estrus behaviors are also very energetically costly [83], both lactation and FPM significantly predicted higher neutrophil counts in zebras; this may reflect an inflammatory response to the potentially injurious activities against mares of suckling young and mating stallions.

Pregnant and lactating mares, and mares with higher FEM in the dry season also had higher tick infestations, despite the fact that ticks are significantly more abundant in the dry season and mares lactate most intensively in the wet. Zebras with higher FEM also had lower anti-PA titers, perhaps indicating reproductive hormone immunomodulatory effects. Lactating zebra and those with higher FEM levels in the wet season also had more intense strongyle infections, likely indicating a periparturient rise in parasitism. Such increases in parasite infections are common around the time of parturition due to energy trade-offs between reproduction and immunity, and the immunosuppressive actions of fluctuating reproductive hormones [84,85].

Despite the potential immunosuppressive effects of pregnancy, parturition, and lactation, however, male zebra had significantly higher strongyle counts, and strongyles were most aggregated in younger animals of both species, the age classes that had the lowest stress hormone levels. While springbok group comparisons for males and females were not significantly different for any parasite, male yearlings again shed significantly more strongyle eggs and *Eimeria* oocysts than did other sex-age groups. These sex aggregations, however, were not related to testosterone levels, as FTM was not included in any final male parasite models. Male-biased parasitism is relatively common in wildlife [86], which has often been attributed to the immunosuppressive effects of testosterone [87]. Our results, however, suggest that Rolff’s application of Bateman’s rule [88,89] (i.e. that females gain fitness through increasing longevity, whereas males gain fitness through increasing mating rates) may be more applicable in this system: he suggested that females are overall more immunocompetent and resistant to infections while males trade off immunity for quicker reproductive fitness, and that this can manifest itself as higher parasite infection intensities in male hosts. The increased parasite infection intensities in younger animals may be due to seasonal reproduction resulting in a pulse of new, immunologically naïve hosts in the wet season, and parasites that have synchronized their life cycles with those of their hosts to ensure that pastures are contaminated with infective stages when transmission is most likely [90]. This decrease in parasite loads with host age also provides evidence for acquired immunity against GI parasites in this system. We also found that more intense strongyle infections predicted higher lymphocyte counts in zebra, despite the fact that lymphocytes were negatively correlated with rainfall; this perhaps correlates with new strongyle infections triggering a memory lymphocyte response in adult hosts, leading to some anti-parasite protection [10,91,92].

### Stress, Pathogens, and Immunity

While stress hormone levels were significantly, positively correlated with several reproductive parameters, increased FGM largely did not correlate with increased pathogen infection intensity either alone or in conjunction with reproductive hormones. In fact, decreased FGM significantly predicted increased strongyles in zebra and increased *Strongyloides* in springbok, and higher strongyle counts in springbok significantly predicted lower FGM levels (note there is no evidence that parasites can directly or indirectly downregulate FGM production; [30]). Strongyle infection intensity in zebras and *Strongyloides* infection intensity in springbok did predict higher FGM levels; this could indicate that being able to acutely mount a small stress response is actually beneficial for animals fighting against new parasite infections. However, the overall magnitude of the negative relationships between FGM and strongyles in zebras was much higher. Thus, while there is some indication here that helminth parasites may correlate with some host stress, there are clearly more complex interactions between parasites and the HPA axis.

This negative or complete lack of association between stress hormones and macroparasites in this study is likely not due to mechanisms of acclimation or decoupling of physical stress and the HPA axis. While acclimation to a stressor can occur when animals experience the same stressor repeatedly, causing the stressor to be perceived to be less noxious and thereby decreasing the GC response [93], acclimation to parasites as predictable stressors is not likely in ENP; pregnancy and mating are also clearly seasonally predictable events in these populations, and stress hormones correlated significantly with these life events. Alternatively, chronic stress can actually cause GCs to decrease with intrinsic control of adrenal function [94]. This is likely not the case here, however, as lowered GCs often represent a highly distressed situation, and zebra and springbok were still capable of secreting significant GCs in response to reproductive and environmental stressors in both seasons.

Overall, then, parasites in this system do not appear to affect host stress levels. This is even more apparent as male yearlings of both species harbored the most parasites of any group, but females of nearly all age-sex comparisons had higher stress levels in zebras. Male adult springbok had nearly significantly higher stress hormone concentrations than did male yearlings, despite yearlings harboring significantly higher parasite intensities. In addition, Turner *et al*. 2012 found that body condition of adult female springbok was significantly, negatively related to strongyle intensity, whereas that of yearlings and adult males was significantly, negatively related to rainfall. Therefore, the springbok age-sex class with the highest condition impact from parasites did not have the highest FGM levels, even in the face of concurrent parturition and lactation. In fact, the only relationship we found between stress, reproduction, and parasites all together was seen in male springbok experiencing both high *Eimeria* intensities and high testosterone concentrations. Thus, only through the interaction with significant amounts of testosterone were parasites correlated with a stress response. While there were significant macroparasite coinfection interactions, none of these interactions were significantly correlated with host stress levels. As increasing GI parasite species richness has been associated with poorer host body condition, the lack of association between coinfection richness in springbok and stress hormones supports the idea that parasites do not cause these hosts significant stress [9].

Given the evidence that GI parasites are capable of causing host pathology [12], immunomodulation [18], and immunosuppression, and that different types of parasites in numerous systems have been found to be strongly correlated with host stress responses in conjunction with parasite effects on host health [95,96], the fact that these parasites persist in ENP hosts with no relationships to chronic stress responses suggests that hosts are tolerant of their parasites (Fig. 6).

Hosts that are good at reducing or clearing parasite burdens are not necessarily the healthiest; eliminating parasites is immunologically costly, and hosts risk immunopathology with vigorous immune responses [97]. In addition, as immunity and reproductive efforts fight for the same resources, increased immune responses, particularly in the face of external resource limitations, can reduce host fecundity. Thus, hosts that can tolerate parasites may have advantages over those that cannot. Tolerance reflects the rate of decline in fitness as parasite burden increases: less fitness decline with increased parasite infection intensity indicates higher parasite tolerance [98]. Tolerance mechanisms are selected for when there are high rates of infection but relatively low virulence [99]. While GI parasites in ENP do affect host condition [13], and likely affect immune function and susceptibility to other pathogens [18], the fact that they are nearly ubiquitous in herbivore hosts suggests that their overall virulence is low (due to transmission-virulence tradeoffs [100]), particularly compared to other pathogens in the system such as *B*. *anthracis*. In addition, while the evolution of resistance should reduce parasite prevalence in host populations, the evolution of tolerance to parasites should have a neutral or positive effect on parasite prevalence [98], as is observed in herbivores in ENP. Thus, it appears that, while GI parasites may influence susceptibility to anthrax and ticks in ENP hosts through immunomodulation [18,31], these parasites do not appear to increase host coinfection susceptibility through stress immunosuppression.

## Conclusions

In summary, we found evidence that stress, reproduction, immunity, and pathogens exhibit strong seasonality in ENP. Most zebra and springbok in ENP are indeed seasonal breeders, with both species experiencing parturition and lactation primarily in the wet season, zebra experiencing most estrus and breeding behaviors in the wet season, and both species experiencing mid-gestation in the hot dry season. Overall immune allocation is highest during the wet season, likely driven, at least in part, by nutritional factors [17]. Stress hormone levels are highest overall in the dry season, though these seasonal signals are stronger in zebras. While peaks in reproductive hormone levels are correlated with immunosuppression, increased parasite infection intensity, and decreased anti-anthrax antibody titers, stress hormone levels are largely unaffected by macroparasite prevalence or infection intensity. Instead, stress hormone levels are most strongly correlated with environmental and reproductive factors, particularly with the demands of mid-gestation. This likely indicates that these hosts are evolutionarily tolerant of their GI parasites, despite the fact that they are not neutral actors in these hosts; parasites do affect body condition and use host resources, hosts do allocate immune resources toward keeping parasite infections in check, and parasites likely cause host immunomodulation and may even increase susceptibility to *B*. *anthracis* in this system. However, parasite tolerance strategies indeed make sense in a system in which macroparasites are nearly ubiquitous but almost never lethal, while pathogens such as *B*. *anthracis* are often lethal but considerably more rare.

## Supporting Information

S1 File Supplemental Information. This file contains supplementary methods for laboratory protocols and statistical analyses, a map of the study region, and tables with information about animal captures, variables and abbreviations, maximal GEE and GLM models, and statistical results for seasonal and sex and age group comparisons.
(ZIP)
