# Supplementary material for: Seasonal Patterns of Hormones, Macroparasites, and Microparasites in Wild African Ungulates: The Interplay among Stress, Reproduction, and Disease
Source: PLoS One. 2015 Apr 15;10(4):e0120800. doi: 10.1371/journal.pone.0120800 (PMC4398380; doi:10.1371/journal.pone.0120800)
Supplement: S1 File — This file contains supplementary methods for laboratory protocols and statistical analyses, a map of the study region, and tables with information about animal captures, variables and abbreviations, maximal GEE and GLM models, and statistical results for seasonal and sex and age group comparisons. (ZIP) [file pone.0120800.s001.zip › S1_SupportingInformationClean 3.docx]

**File S1: Supporting Information**

**Supplementary Methods**

**Gastrointestinal Parasites**

Zebra in ENP experience infections with helminths in the order Rhabditia, suborder Strongylida, primarily within the superfamily Strongyloidea, and family Strongylidae; this group contains both the “large strongyles” (spp. in the subfamily Strongylinae) and the “small strongyles” (spp. in the subfamily Cyathstominae). These parasites are oviparous and exhibit a direct life cycle with three, free-living larval stages. The first two moults to the infectious L3 stage occur over one to two weeks, after which L3’s are ingested by herbivore hosts [1]. The first two free-living stages are highly susceptible to desiccation, and develop more quickly and survive for longer in relatively warm temperatures and humid conditions [2].

Springbok typically experience infections with strongyles in the superfamily Trichostrongyloidea [3]. While previous studies of these parasites in ENP have not distinguished these parasites beyond the family level, evidence from springbok studies in South Africa suggest that *Trichostrongylus* and *Paracooperia* species (family Trichostrongylidae, subfamily Trichostrongylidae) and *Cooperia* species (family Cooperiidae) are the dominant intestinal strongyles in springbok in this region [4–6]. Springbok in ENP also experience intestinal infections with *Strongyloides* species, nematodes in the order Rhabditida and family Strongyloididae [6,7]. Only parasitic adult females reside within host guts; these worms reproduce parthenogenetically, shedding eggs in host feces [8]. In the external environment, eggs hatch into L1 larvae, develop to the infective L3 stage, and penetrate host skin.

Springbok in ENP also experience infections with coccidian parasites in the genus *Eimeria* (class Coccidia; order Eucoccidiorida; family Eimeriida) [6]. *Eimeria* are typically very host-specific, and three species of this parasite have been identified in ENP springbok [9]. *Eimeria* are transmitted fecal-orally between hosts, via an environmental stage. Oocysts are shed in host feces, undergo development (sporulation), and become infectious to new hosts within approximately one week [10]. While oocysts are fairly hardy, they require moisture and lower temperatures for long-term environmental survival [10].

**Rain Group Determination**

Histograms of cumulative rainfall prior to each individual sampling event for captured zebras revealed a distinct bimodal distribution in the individually-linked rainfall, regardless of qualitatively named seasons (Fig. B). We thus chose, when carrying out "seasonal" comparisons within and among individuals, to group rainfall by quantitative categories (as described below) rather than by qualitative seasons. This made both ecological and biological sense: those “wet season” animals sampled late in April and the beginning of May (the typical start of the cold dry season) accordingly fell into the lower rainfall group; and external moisture greatly influences the within-host development and egg-producing activity of GI parasites [11,12]. The rain groups were thus for captured zebra: “wet season,” the high rainfall group, containing individuals that had experienced > 200mm rainfall two months prior to sampling; and “dry season,” the low rainfall group, containing individual samplings connected with < 100mm rainfall in the two months prior.

We divided non-captured (NC) zebra and springbok into similar rain group categories. While individually-experienced rainfall was not as strongly bimodal for these samples as it was for captured zebra, all "wet season" rainfall values below 105mm of rain were experienced by those animals sampled late April and after. The rainfall groups were thus for NC zebra and springbok: “wet season” for those experiencing >120mm rainfall in the two months prior to sampling; and “dry season” for those experiencing <120mm rainfall in the two months prior (there were no rainfall values between 105 and 122mm).

**Hematocrit, Blood Smears, and Counts with Differentials**

Whole blood for hematocrit (HCT) and neutrophil and lymphocyte counts was collected into Vacutainers (Becton Dickinson, Franklin Lakes, NJ) containing EDTA anticoagulant. We measured HCT, a measure of percent of red blood cells per volume of blood, within five hours of whole blood collection using heparinized capillary tubes and a micro-hematocrit card style reader (StatSpin, Westwood, MA).

To determine neutrophil and lymphocyte concentrations, we created thin blood smears on glass slides, fixed them with methanol, and stained them with Diff-Quik (Dade Behring, Deerfield, IL). We performed manual total white blood cell (WBC) counts using a compound microscope; we counted cells in ten fields at 40x magnification and multiplied mean cell count per field by 1600 (magnification^2^) to obtain total WBCs per μl of blood. We did differential counts by determining the percent of each of the most common WBC types (neutrophils, monocytes, lymphocytes, eosinophils) in 200 WBCs counted at 40x and multiplying this by total WBC concentration to obtain numbers of neutrophils or lymphocytes per μl of blood. All counts were done in duplicate and averaged.

**Anti-Anthrax Antibody Concentrations**

We used wildtype *Bacillus anthracis* protective antigen (PA) as coating antigen at a concentration of 0.375μl per well. We made serial, twofold dilutions to the ends of rows in duplicate for all samples and negative controls, starting at a dilution of 1:4 and ending at 1:8192 and ran a duplicate negative control full titration series on each ELISA plate. We used goat-anti-horse IgG-heavy and light chain horseradish peroxidase (HRP) conjugate (Bethyl Laboratories, Montgomery, TX) and added TMB substrate (Kirkegaard & Perry Laboratories; Gaithersburg, MD), stopping the reaction with 2N sulfuric acid. We read well absorbance as optical density (OD) at 450nm on a SpectraMax M2 Microplate Reader using SoftMax Pro software v5.3 (Molecular Devices; Sunnyvale, CA). As we had no known, titrated standards to establish a standard curve, we determined the endpoint titers as the log_2_ of the last sample dilution at which the mean OD for that sample at that dilution was greater than the mean OD for all negative controls at that dilution, buffered by a 95% confidence interval determined by the inter-duplicate error at that dilution, across all samples analyzed.

**Modified McMaster Protocol**

We used a modified McMaster method to count parasite eggs in feces [13]. Briefly, we combined 4g of homogenized fecal matter with 56ml of a saturated NaCl solution (specific gravity 1.2), removed any large debris with a strainer, and obtained a homogenized filtrate. We placed an aliquot of filtrate into each chamber of a McMaster slide and counted the number of eggs observed in each chamber using a compound microscope at 10x magnification. We obtained a measure of eggs per gram of feces by adding the number of eggs for both chambers and multiplying by 50.

Fecal egg counts (FECs) provide an accurate estimate of how the input of parasite eggs into the environment varies with other factors of interest [7]. While the actual relationship between fecal egg count and total nematode burden within a host is of unknown specificity and sensitivity, these counts provide a nonlethal and often noninvasive method for estimating these infection burdens [14,15]. In addition, we previously found that fecal water content had no effect on seasonal and age-related patterns in strongyle egg counts, thus increasing our confidence regarding the overall accuracy of this measurement [16].

**Hormone Metabolite Measurement**

We manually mixed samples within bags prior to selecting a subsample for powdering with an electric coffee grinder. After removing large particulate matter, we selected roughly half of each powdered sample for determining dry weight; samples were dried in foil packets in a drying oven at 80-100°C for 24-48 hours. For extractions, we selected 1g (weighed to the nearest 0.001g) of non-dried, powdered sample, added 5ml methanol, and vortexed for 30-60 minutes. We then centrifuged samples at 3000rpm for 15 minutes and stored supernatants at -20°C prior to analysis.

Concentrations of cortisol metabolite immunoreactivity in fecal extracts were quantified using a double antibody ^125^I-corticosterone radioimmunoassay (RIA) (MP Biomedicals, Santa Ana, CA) validated for zebra and other antelope species [17] and used to assess translocation stress in zebra [18]. No corticoid immunoreactivity was detected in zebra or springbok fecal extracts using a ^125^I-cortisol RIA (Coat-a-Count, Diagnostic Products, CA, USA). We quantified concentrations of progestin metabolite immunoreactivity in fecal extracts using an ^125^I- antibody coated tube RIA (Diagnostic Systems Laboratories, Webster, TX) [122]. Assays such as this one with cross-reactivity to a wide range of progesterone metabolites have been used to successfully quantify progesterone in a wide range of species [126], and previous studies have found strong correlations between serum progesterone concentration and fecal progesterone metabolites [20]. We quantified concentrations of estrogen metabolite immunoreactivity in fecal extracts using an ^125^I- double antibody RIA (Diagnostic Systems Laboratories). Previous studies have found significant correlations between serum estrogen concentrations and fecal estrogen metabolite levels [20]. We quantified concentrations of testosterone metabolite immunoreactivity in fecal extracts were quantified using an ^125^I- antibody coated tube RIA (Immunotech, Marseille, France). Previous studies have found significant positive correlations between fecal testosterone metabolites and serum testosterone concentration [21]. Fifty microliters, between 5 and 20μl, and between 25 and 50μl of fecal extract for cortisol and testosterone, progesterone, and estrogen, respectively, were added to steroid diluent and then assayed according to the manufacturer’s instructions. Parallelism between serial dilutions of fecal extracts for both zebra and springbok and the standard curves were obtained, validating use of the assays in each species. Assay detection limits, sensitivities, and assay variations are listed in Table K in S1 File.

We used estrogen peak concentrations to determine when animals were in mid-gestation. To determine whether using rain groups rather than nominal seasons shifted our interpretation of timing of reproductive effects (i.e. because we were examining gestation not only in concert with rainfall, but also in terms of strict temporal physiological cycles), we examined separately those individuals who had been captured in a nominal season but were reassigned to a different rain group season. Thirty-two late “wet season” zebra captures were reassigned to the dry season rain group. Of these, only one animal was determined to be pregnant through palpation, and only seven had high FEM concentrations concordant with mid-gestation (greater than 10000 pg/ml). Thus, our determining that the majority of pregnant ENP mares were in mid-gestation in the middle of the hot dry season was potentially only confounded by eight samples; as the other potentially confounding samples indicated negative pregnancies and low FEM concentrations in the dry season, the fact that this population experienced significantly higher FEM concentrations in the dry season compared to the wet is all the more supported (Fig. 2). For our NC zebra, only three animals were sampled in the late “wet season” but assigned to the dry season rain group, and all three had FEM concentrations that were not indicative of mid-gestation. No springbok samples were discordant between nominal seasons and rain groups.

**Multiple Imputation of Missing Data**

Multiple imputation is most often used in human public health studies in which some data are missing for individuals sampled repeatedly over time [e.g. 141]. Comparisons of analyses using multiply imputed datasets versus complete case analysis (CCA), in which cases with any missing data are eliminated from the analysis, have found that MI produces much less biased results. This is true when both small and large amounts of data points are missing [23]. In addition, CCA has been found to be appropriate only when data are known to be absolutely missing completely at random (MCAR); because there are often underlying, potentially unobserved causes for missing data, using CCA is often suboptimal [24,25].

For imputation, we used the Multiple Imputation by Chained Equations (MICE) method with the 'mice' package [26] in R v2.15.2 [27]. This method specifies the imputation model for each variable by building, and iterating over, a set of conditional densities for each variable. We built our predictor matrix by first using all variables in this study [24], and then refined the predictor matrix for each variable to avoid collinearity. We preserved all data transformations by passively imputing each transformed variable linked to its original variable [26]. We validated our imputations by confirming convergence, examining density plots and strip plots to ensure that imputed values overlapped existing data, and comparing distributions of observed versus imputed data based on propensity scores [26].

**Generalized Estimating Equation and General Linear Models**

For captured zebra GEE, we square-root transformed GI parasite (GIP), ectoparasite, lymphocyte, and age response variables, and log transformed neutrophil and FGM response variables for our GEE models to deal with overdispersion. In addition, we used log-transformed FGM in the PA model and square root-transformed GIP in our lymphocyte model (Table D in S1 File). These transformations did not affect model selection or significance, but improved residual patterns and normality. As these transformations affect the direct interpretation of coefficient estimates, interpretations here are based solely on sign and significance of coefficients to determine predictors of the response variables and the directions of these effects.

For NC Zebra GLM, we log transformed FEM and fourth-root transformed FPM in all models to improve model residual patterns. As for the NC zebra GLM models, we transformed certain explanatory variables (GIP, GIS, and GIC) in all springbok models to improve model residual patterns (Table F in S1 File).

**Supplemental Information References**

1. Durette-Desset M-C, Beveridge I, Spratt D (1994) The origins and evolutionary expansion of the Strongylida (Nematoda). Int J Parasitol 24: 1139–1165.

2. Nielsen MK, Kaplan RM, Thamsborg SM, Monrad J, Olsen SN (2007) Climatic influences on development and survival of free-living stages of equine strongyles: implications for worm control strategies and managing anthelmintic resistance. Vet J 174: 23–32. Available: http://www.ncbi.nlm.nih.gov/pubmed/16815051. Accessed 19 July 2011.

3. Bowman DD (2003) Georgis’ parasitology for veterinarians, 8th edition. 8th editio. Philadelphia, PA: W.B. Saunders.

4. Horak IG, Meltzer DGA, de Vos V (1982) Helminth and arthropod parasites of springbok, Antidorcas marsupialis, in the Transvaal and Western Cape Province. Onderstepoort J Vet Res 49: 7–10.

5. De Villiers IL, Liversidge R, Reinecke RK (1985) Arthropods and helminths in springbok (Antidorcas marsupialis) at Benfontein, Kimberley. Onderstepoort J Vet Res 52: 1–11.

6. Jain E, Bairoch A, Duvaud S, Phan I, Redasch N, et al. (2009) Infrastructure for the life sciences: design and implementation of the UniProt website. BMC Bioinformatics. Available: http://www.biomedcentral.com/1471-2105/10/136.

7. Turner WC, Getz WM (2010) Seasonal and demographic factors influencing gastrointestinal parasitism in ungulates of Etosha National Park. J Wildl Dis 46: 1108–1119.

8. Viney ME, Lok JB (2007) Strongyloides spp. In: Community TC elegans R, editor. WormBook. pp. 1–15. Available: http://www.wormbook.org. Accessed 28 February 2013.

9. Turner WC (2009) The ecology of orally ingested parasites in ungulates of Etosha National Park University of California, Berkeley.

10. Daugschies A, Najdrowski M (2005) Eimeriosis in cattle: current understanding. J Vet Med B 52: 417–427. Available: http://www.ncbi.nlm.nih.gov/pubmed/16364016.

11. Horak IG (1981) The seasonal incidence of the major nematode genera recovered from sheep, cattle, impala and blesbok in the Transvaal. J S Afr Vet Assoc 52: 213–223.

12. Shaw JL (1988) Arrested development of Trichostrongylus tenuis as third stage larvae in red grouse. Res Vet Sci 45: 256–258.

13. Gibbons LM, Jacobs DE, Fox MT, Hansen J (2005) McMaster egg counting technique. R Vet Coll Agric Organ United Nations Guid to Vet Diagnostic Parasitol. Available: http://www.rvc.ac.uk/Review/Parasitology/EggCount/Purpose.htm.

14. Stear MJ, Bishop SC, Duncan JL, McKellar QA, Murray M (1995) The repeatability of faecal egg counts, peripheral eosinophil counts, and plasma pepsinogen concentrations during deliberate infections with Ostertagia circumcincta. Int J Parasitol 25: 375–380.

15. Seivwright LJ, Redpath SM, Mougeot F, Watt L, Hudson PJ (2004) Faecal egg counts provide a reliable measure of <I>Trichostrongylus tenuis</I> intensities in free-living red grouse <I>Lagopus lagopus scoticus</I>. J Helminthol 78: 69–76. Available: http://journals.cambridge.org/abstract_S0022149X04000113. Accessed 4 November 2012.

16. Turner WC, Cizauskas C a, Getz WM (2010) Variation in faecal water content may confound estimates of gastro-intestinal parasite intensity in wild African herbivores. J Helminthol 84: 99–105. Available: http://www.pubmedcentral.nih.gov/articlerender.fcgi?artid=2833100&tool=pmcentrez&rendertype=abstract. Accessed 1 September 2014.

17. Chinnadurai SK, Millspaugh JJ, Matthews WS, Canter K, Slotow R, et al. (2008) Validation of fecal glucocorticoid metabolite assays for South African herbivores. J Wildl Manage 73. doi:10.2193/2008-430.

18. Franceschini MD, Rubenstein DI, Low B, Romero LM (2008) Fecal glucocorticoid metabolite analysis as an indicator of stress during translocation and acclimation in an endangered large mammal, the Grevy’s zebra. Anim Conserv 11: 263–269. Available: http://doi.wiley.com/10.1111/j.1469-1795.2008.00175.x. Accessed 6 March 2013.

19. Sanders H, Rajamahendran R, Burton B (1994) The development of a simple fecal immunoreactive progestin assay to monitor reproductive function in swine. Can Vet J 35: 355–358. Available: http://www.pubmedcentral.nih.gov/articlerender.fcgi?artid=1686265&tool=pmcentrez&rendertype=abstract.

20. Capezzuto A, Chelini MOM, Felippe ECG, Oliveira CA (2008) Correlation between serum and fecal concentrations of reproductive steroids throughout gestation in goats. Anim Reprod Sci 103: 78–86. Available: http://www.ncbi.nlm.nih.gov/pubmed/17156948. Accessed 4 June 2013.

21. Chang G-R, Hsu S-H, Hsu TH, Yang C-C, Chan F-T, et al. (2009) Seasonal influence on fecal immunoreactive testosterone concentrations of male Formosan black bears (Ursus thibetanus formosanus). Eur J Wildl Res 55: 203–208.

22. Johansen NB, Vistisen D, Brunner EJ, Tabák AG, Shipley MJ, et al. (2012) Determinants of aortic stiffness: 16-year follow-up of the Whitehall II study. PLoS One 7: e37165. Available: http://www.pubmedcentral.nih.gov/articlerender.fcgi?artid=3358295&tool=pmcentrez&rendertype=abstract. Accessed 20 December 2012.

23. Van der Heijden GJMG, Donders a RT, Stijnen T, Moons KGM (2006) Imputation of missing values is superior to complete case analysis and the missing-indicator method in multivariable diagnostic research: a clinical example. J Clin Epidemiol 59: 1102–1109. Available: http://www.ncbi.nlm.nih.gov/pubmed/16980151. Accessed 29 November 2012.

24. Rubin DB (1996) Multiple imputation after 18+ years. J Am Stat Assoc 91: 473–489.

25. Vergouw D, Heymans MW, van der Windt D a WM, Foster NE, Dunn KM, et al. (2012) Missing data and imputation: a practical illustration in a prognostic study on low back pain. J Manipulative Physiol Ther 35: 464–471. Available: http://www.ncbi.nlm.nih.gov/pubmed/22964020. Accessed 20 December 2012.

26. Van Buuren S, Groothuis-Oudshoorn K (2011) mice: Multivariate imputation by chained equations in R. J Stat Softw 45: 1–67.

27. Team R (2013) R Development Core Team. R A Lang Environ Stat Comput. Available: http://www.mendeley.com/research/r-language-environment-statistical-computing-96/\npapers2://publication/uuid/A1207DAB-22D3-4A04-82FB-D4DD5AD57C28.

**Supplemental Information Legends**

**Fig. A.** Etosha National Park in northern Namibia. The Etosha Ecological Institute is located in Okaukuejo in the center of the park; the majority of animal sampling for this study occurred in the nearby surrounding area, within a radius of approximately 20km (in the plains outside of the salt pans). During drier seasons, some sampling took place up to 100km to the east of Okaukuejo, around the Halali plains, and 15km south of Okaukuejo.

**Fig. B.** **a. Mean (±SE) monthly Okaukuejo rainfall from 1974-2010.**

**b. Cumulative rainfall 2 months prior to each zebra capture (Rain2), for all captures over all seasons.** The total rainfall in the 60 days prior to capture was determined for each individual zebra capture event, and that number was assigned to that individual-capture as its associated rainfall amount. While we sampled animals in nominally "wet" or "dry" seasons, we saw a clear bimodal pattern in rainfall amounts that did not necessarily align with seasons. This is particularly noticeable in gray bars: Rain2 experienced by animals sampled in the nominal wet season. We therefore used rainfall amounts to assign each individual-capture to a rain season: “wet season,” the high rainfall group, containing individuals that had experienced > 200mm rainfall two months prior to sampling; and “dry season,” the low rainfall group, containing individual samplings connected with < 100mm rainfall in the two months prior. Black bars: Rain2 experienced by animals sampled in the nominal dry season.

**Supplemental Information Tables**

**Table A.** **Zebra capture seasons, timing, animals involved, and samples taken.**

| **CS** | **NS** | **Date**  **(Mo/Yr)** | **Blood** | **Feces** | **Ticks** |
| --- | --- | --- | --- | --- | --- |
| S1 | Wet | 3-4/08 | 45(45,0) | 38(38,0) | 45(45,0) |
| S2 | Dry | 10-11/08 | 36(14,22) | 29(17,12) | 18(0,18) |
| S3 | Wet | 4-5/09 | 35(6, 29) | 32(4,28) | 30(5,25) |
| S4 | Dry | 9-11/09 | 13(4,9) | 10(3,7) | 13(4,9) |
| S5 | Dry | 8/10 | 25(0,25) | 14(0,14) | 19(0,19) |
| **Totals** |  |  | 154(69,85) | 123(62,61) | 125(54,71) |

"CS" is Capture Season and "NS" is Nominal Season.

Data refer to Total# (#New, #Resampled), where "New" refers to new individuals and their samples and "Resampled" refers to animals resampled at least once in that season and their corresponding samples collected. "Ticks" refers to the number of zebras sampled for total tick burden.

**Table B.** **A. Number of zebra captured in each season for first captures only, grouped by seasons. B. Number of zebra captured in each season for paired recaptured only, grouped by seasons.**

| **A.** | **Capture Season** | **Cap1Wet** | **Cap1Dry** | **B.** | **Capture Season** | **Cap1Wet** | **Cap2Dry** |
| --- | --- | --- | --- | --- | --- | --- | --- |
|  | S1 | 45 | 0 |  | S1 | 32 | 0 |
|  | S2 | 0 | 14 |  | S2 | 0 | 23 |
|  | S3 | 0 | 6 |  | S3 | 0 | 8 |
|  | S4 | 0 | 4 |  | S4 | 0 | 1 |
|  | S5 | 0 | 0 |  | S5 | 0 | 0 |

There were no resampled animals that fell into Cap1Dry or Cap2Wet groups.

Cap1 is capture 1; Cap2 is capture 2 for the same individual; Wet is the higher rain group (experience of cumulative rainfall >200mm over the two months prior to capture); Dry is the lower rain group (experience of cumulative rainfall <100mm over the two month prior to sampling).

**Table C. List of variables used in models, with their abbreviations and descriptions.**

| **Variable** | **Abbreviation** | **Subjects** | **Description** |
| --- | --- | --- | --- |
| Cumulative rain 2 months prior | Rain2 | All | Rain (mm) experienced by an individual in 60 days prior to a capture event |
| Sex | Sex; M or F | All | Male (M) or female (F) |
| Individual age | Age | Captured (C) Zebras | Age (days) at a sampling event, determined first by dental wear |
| Age Class | AgeC; A or Y | NC zebras; springbok | Adults (>2 years old; A) or yearlings (<2 years old; Y) |
| GI Strongyle parasite burden | GIP, or GIPsqrt when square root transformed | All | GI strongyle helminth infection intensity (nematode eggs/g of feces) |
| GI coccidia burden | GIC, GICsqrt, or GIC4 when 4^th^ root transformed | Springbok | GI coccidian infection intensity (*Eimeria* oocysts/g of feces) |
| GI *Strongyloides* burden | GIS, GISsqrt, or GIS4 | Springbok | GI *Strongyloides* helminth infection intensity (nematode eggs/g of feces) |
| Sublethal anthrax exposure | log2PA or PA | C zebras | Anti-PA antibody titer as measured in log_2_ of final dilution (log2PA) or as presence or absence of a titer (PA) |
| Ectoparasite burden | Ecto, or Ectosqrt | C zebras | Total number of ticks |
| Hematocrit | HCT or HCTsqrt | C zebras | % of blood volume comprised of red blood cells |
| White blood cell count | WBC, or WBCsqrt | C zebras | Number of total white blood cells /μl of blood |
| Neutrophil count | Neut, or logNeut when log_10_ transformed | C zebras | Number of neutrophils/μl of blood |
| Lymphocyte count | Lymph, or Lymphsqrt | C zebras | Number of lymphocytes/μl of blood |
| Fecal glucocorticoid metabolites | FGM, logFGM, or FGM4 | All | Glucocorticoid metabolite concentration in feces (ng/g of dry fecal weight) |
| Fecal progesterone | FPM, FPMsqrt, or FPM4 | All (females) | Progesterone metabolite concentration in feces (ng/g of dry fecal weight) |
| Fecal estrogen | FEM, logFEM, or FEM4 | All (females) | Estrogen metabolite concentration in feces (pg/g of dry fecal weight) |
| Fecal testosterone | FTM | NC zebra, springbok (males) | Testosterone metabolite concentration in feces (ng/g dry fecal weight) |
| Foal presence | Foal | C zebras | Presence or absence of a foal with a mother |
| Pregnancy | Preg | C zebras | Obvious presence (2), obvious absence (0), and likely but unconfirmed presence (1) of pregnancy |
| Lactating | Lact | C zebras | Lactating milk (2), watery discharge (1), or no lactation (0) |

**Table D.** **Maximal generalized estimating equation models evaluated for captured zebras.**

| **Pathogen Models** |  |  | |
| --- | --- | --- | --- |
| GIPsqrt | ~ | Rain2 + Neut + Lymph + HCT + FGM + FPM + FEM + log2PA + Ecto + Age + Foal + Preg + Lact |  |
| PA | ~ | Rain2 + Neut + Lymph + HCT + logFGM + FPM + FEM + GIP + Ecto + Age + Foal + Preg + Lact |  |
| Ectosqrt | ~ | Rain2 + Neut + Lymph + HCT + FGM + FPM + FEM + log2PA + GIP + Age + Foal + Preg + Lact |  |
| **Immune Models** |  |  |  |
| logNeut | ~ | Rain2 + Lymph + HCT + FGM + FPM + FEM + log2PA + GIP + Ecto + Age + Foal + Preg + Lact |  |
| sqrtLymph | ~ | Rain2 + logNeut + HCT + FGM + FPM + FEM + log2PA + GIPsqrt + Ecto + Age + Foal + Preg + Lact |  |
| **Hormone Model** |  |  |  |
| logFGM | ~ | Rain2 + Neut + Lymph + HCT + FPM + FEM + log2PA + GIP + Ecto + Age + Foal + Preg + Lact |  |
| **Age Model** |  |  |  |
| Agesqrt | ~ | Neut + Lymph + HCT + FGM + FPM + FEM + log2PA + GIP + Ecto + Foal + Preg + Lact |  |

**Table E.** **Maximal generalized linear models evaluated for non-captured zebras.**

| **Strongyle Helminth Models** | | |  |  |
| --- | --- | --- | --- | --- |
| GIP | ~ | Rain2 + AgeC + Sex + FGM | | |
| GIP | ~ | Rain2 + AgeC + Sex + FGM + FPM4 + logFEM | | |
| GIP (males only) | ~ | Rain2 + FGM + FTM | | |
| **Hormone Models** |  |  | | |
| FGM | ~ | Rain2 + AgeC + Sex + GIP | | |
| FGM | ~ | Rain2 + AgeC + Sex + GIP + FPM4 + logFEM | | |
| FGM (males only) | ~ | Rain2 + GIP + FTM | | |

All GIP models use the negative binomial distribution with a log link, while all FGM models use the gamma distribution with an inverse link.

**Table F.** **Maximal generalized linear models and zero-inflated models evaluated for springbok.**

| **Strongyle Helminth Models** | | |  |  | | |  |
| --- | --- | --- | --- | --- | --- | --- | --- |
| GIP | ~ | Rain2 + AgeC + Sex + GISsqrt + GIC4 + FGM | | | | |  |
| GIP | ~ | Rain2 + AgeC + Sex + GISsqrt + GIC4 + FGM + FPM4 + logFEM | | | | |  |
| GIP (males only) | ~ | Rain2 + GISsqrt + GIC4 + FGM + FTM | | | | |  |
| ***Strongyloides* Helminth Models** | | | | |  |  | |
| GIS | ~ | Rain2 + AgeC + Sex + GIPsqrt + GIC4 + FGM | | | | |  |
| GIS | ~ | Rain2 + AgeC + Sex + GIPsqrt + GIC4 + FGM + FPM + FEM | | | | |  |
| GIS (males only) | ~ | Rain2 + GIPsqrt + GIC4 + FGM + FTM | | | | |  |
| ***Eimeria* Models** |  |  | | | | |  |
| GIC | ~ | Rain2 + AgeC + Sex + GIPsqrt + GISsqrt + FGM | | | | |  |
| GIC | ~ | Rain2 + AgeC + Sex + GIPsqrt + GISsqrt + FGM + FPM + FEM | | | | |  |
| GIC (males only) | ~ | Rain2 + GIPsqrt + GISsqrt + FGM + FTM | | | | |  |
| **Hormone Models** |  |  | | | | |  |
| FGM | ~ | Rain2 + AgeC + Sex + GIPsqrt + GISsqrt + GIC4 | | | | |  |
| FGM | ~ | Rain2 + AgeC + Sex + GIPsqrt + GISsqrt + GIC4 + FPM + FEM | | | | |  |
| FGM (males only) | ~ | Rain2 + GIPsqrt + GISsqrt + GIC4 + FTM | | | | |  |

All GIP and GIC generalized linear models use the negative binomial distribution with a log link, while all FGM generalized linear models use the gamma distribution with an inverse link. All GIS models are zero inflated negative binomial models; counts portions of the models use the negative binomial distribution with a log link, while zero-inflated portions of the models use a binomial distribution with a logit link.

**Table G.** **Results of two-tailed Welch's *t* tests and Wilcoxon rank sum tests comparing variables between rain groups for first captured zebra samplings (unique animals) only.**

| **Variable** | **df** | ***t* or *U*** | ***p*-value before correction** | **Holm's corrected *p*-value** | **Mean Difference^#^** | **Higher Group** |
| --- | --- | --- | --- | --- | --- | --- |
| **GIPsqrt** | 54.5 | 5.07 | **0.000***** | **0.000***** | 1807 | **Wet** |
| log2PA |  | 561^+^ | 0.117 | 0.490 | 1.18 | Wet |
| Ecto |  | 420^+^ | 0.604 | 1.000 | 0.23 | Dry |
| **WBCsqrt** | 57.7 | 2.80 | **0.007**** | **0.042*** | 1213 | **Wet** |
| **logNeut** | 54.9 | 3.41 | **0.001***** | **0.010**** | 870 | **Wet** |
| Lymphsqrt | 49.1 | 0.70 | 0.488 | 1.000 | 148 | Wet |
| **HCTsqrt** | 46.1 | 3.15 | **0.003**** | **0.020*** | 2.72 | **Wet** |
| logFGM | 46.7 | -1.69 | 0.098. | 0.490 | 357 | Dry |
| FPMsqrt | 53.0 | 0.90 | 0.371 | 1.000 | 175 | Wet |
| **logFEM** | 56.6 | -8.30 | **0.000***** | **0.000***** | 6592 | **Dry** |

Significant tests (*p*< 0.05) are in bold.

*N*=38 for all Wet Season variables and 24 for all Dry Season.

^+^ are Wilcoxon rank sum test results, using the test statistic *U*.

^#^ Mean differences are differences between non-transformed means in the two rain groups for all

variables. Units for mean differences are eggs per gram of feces for GIP; log_2_ titer for log_2_PA;

number of ticks for Ecto; cells/μl of blood for WBC, Neut, and Lymph; percent for HCT; ng/g of fecal

dry weight for FGM and FPM; and pg/g of fecal dry weight for FPM.

. *p*<0.1; **p*<0.05; ***p*<0.01; ****p*<0.001

**Table H.** **Results of paired two-tailed *t* tests and Wilcoxon signed rank tests comparing variables between rain groups for first and second captured zebra samplings of the same individuals.**

| **Variable** | ***t* or *T*** | ***p*-value before correction** | **Holm's corrected *p*-value** | **Mean Difference^#^** | **Higher Group** |
| --- | --- | --- | --- | --- | --- |
| **GIPsqrt** | 4.88 | **0.000***** | **0.000***** | 1926 | **Wet** |
| log2PA | 136^+^ | 0.698 | 1.000 | 0.18 | Dry |
| **Ecto** | 48.5^+^ | **0.004**** | **0.019*** | 2.61 | **Dry** |
| **WBCsqrt** | 3.59 | **0.001***** | **0.010**** | 1836 | **Wet** |
| **logNeut** | 3.54 | **0.002**** | **0.010**** | 1046 | **Wet** |
| **Lymphsqrt** | 3.25 | **0.003**** | **0.019*** | 629 | **Wet** |
| HCTsqrt | 2.47 | **0.020*** | 0.080. | 2.61 | Wet |
| logFGM | -2.06 | **0.049*** | 0.147 | 649 | Dry |
| FPMsqrt | -0.62 | 0.539 | 1.000 | 118 | Dry |
| **logFEM** | -5.67 | **0.000***** | **0.000***** | 5194 | **Dry** |

Significant tests (*p*<0.05) are in bold.

*N*=28 (and df=27) for all variables in both rain groups.

^+^ are Wilcoxon signed rank test results, using the test statistic *T*.

^#^ Mean differences are differences between non-transformed means in the two rain groups for all

variables. Units for mean differences are eggs per gram of feces for GIP; log_2_ titer for log_2_PA;

number of ticks for Ecto; cells/μl of blood for WBC, Neut, and Lymph; percent for HCT; ng/g of fecal

dry weight for FGM and FPM; and pg/g of fecal dry weight for FPM.

. *p*<0.1; **p*<0.05; ***p*<0.01; ****p*<0.001

**Table I.** **Results of Tukey's HSD tests or two-tailed Wilcoxon rank sum tests comparing variables between rain, sex, and age groups for non-captured zebra.**

| **Variable** | **Comparison** | ***N*** | ***U***^+^ | **Tukey's or Holm's corrected *p-*value** | **Mean Difference^#^** | **Higher Group** |
| --- | --- | --- | --- | --- | --- | --- |
| GIPsqrt | **Seasons** | 169, 143 |  | **0.001***** | 479 | **Wet** |
|  | **Sexes** | 158, 154 |  | **0.003**** | 473 | **males** |
|  | Ages | 33, 279 |  | 0.449 | 353 | yearlings |
|  | **MY, FY^@^** | 22, 11 |  | **0.012*** | 1764 | **MY** |
|  | **MY, FA** | 22, 143 |  | **0.012*** | 1095 | **MY** |
| FGM | **Seasons** | 165, 137 | 15910 | **0.000***** | 693 | **Wet** |
|  | **Sexes** | 154, 148 | 8979 | **0.016*** | 320 | **females** |
|  | **Ages** | 31, 271 | 3029 | **0.022*** | 369 | **adults** |
|  | **MY, FY** | 22, 9 | 44 | **0.022*** | 960 | **FY** |
|  | **MY, MA** | 22, 132 | 922 | **0.019*** | 528 | **MA** |
|  | **MY, FA** | 22, 139 | 733 | **0.000***** | 761 | **FA** |
|  | **WetM, DryM** | 80, 74 | 3901 | **0.006**** | 565 | **WetM** |
|  | **WetM, WetF** | 80, 85 | 2680 | **0.047*** | 388 | **WetF** |
|  | **WetM, DryF** | 80, 63 | 3042 | **0.047*** | 408 | **WetM** |
|  | **DryM, WetF** | 74, 85 | 1301 | **0.000***** | 953 | **WetF** |
|  | **WetF, DryF** | 85, 63 | 3978 | **0.000***** | 796 | **WetF** |
|  | **WetY, WetA** | 20, 145 | 881 | **0.029*** | 618 | **WetA** |
|  | **DryY, WetA** | 11, 145 | 346 | **0.014*** | 906 | **WetA** |
|  | **WetA, DryA** | 145, 126 | 13338 | **0.000***** | 756 | **WetA** |
| FPM | **Seasons** | 103, 60 | 3901 | **0.016*** | 310 | **Wet** |
|  | **Sexes** | 59, 104 | 2337 | **0.023*** | 295 | **females** |
|  | **Ages** | 9, 154 | 377 | **0.023*** | 494 | **adults** |
|  | **DryM, WetF** | 26, 70 | 500 | **0.003**** | 529 | **WetF** |
| FEM | **Seasons** | 103, 60 | 1739 | **0.000***** | 1961 | **Dry** |
|  | Sexes | 59, 104 | 3062 | 0.984 | 1574 | females |
|  | Ages | 9, 154 | 424 | 0.102 | 1886 | adults |
|  | **WetM, DryF** | 33, 34 | 288 | **0.002**** | 3962 | **DryF** |
|  | **DryM, WetF** | 26, 70 | 1225 | **0.038*** | 235 | **DryM** |
|  | DryM, DryF | 26, 34 | 309 | 0.143 | 3728 | DryF |
|  | **WetF, DryF** | 70, 34 | 557 | **0.000***** | 3395 | **DryF** |
| FTMsqrt | Seasons | 64, 39 |  | 0.535 | 2.18 | Dry |
|  | **Ages** | 15, 88 |  | **0.000***** | 22.4 | **adults** |

Significant tests (*p*< 0.05) are in bold.

For non-normal variables, interaction effect comparisons were only explored with Wilcoxon rank sum tests if preliminary explorations with ANOVA and Tukey HSD tests revealed a *p*<0.1. For normalized variables examined with Type III ANOVAs and Tukey HSD tests, only significant interaction comparisons (*p*<0.05) are listed here.

N's are listed as males always first; then yearlings first; then Wet Season first.

^@^MY=male yearlings; MA=male adults; FY=female yearlings; FA=female adults

^+^ Comparisons listed with a *U* test statistic were analyzed with Wilcoxon rank sum tests. *p* values for

these tests were adjusted for familywise error rates using the Holm's Bonferroni correction.

^#^ Mean differences are differences between non-transformed means for all variables. Units for mean

differences are eggs per gram of feces for GIP; ng/g of fecal dry weight for FGM and FPM; and pg/g

of fecal dry weight for FPM.

. *p*<0.1; **p*<0.05; ***p*<0.01; ****p*<0.001

**Table J.** **Results of Tukey's HSD tests or two-tailed Wilcoxon rank sum tests comparing variables between rain, sex, and age groups for springbok.**

| **Variable** | **Comparison** | ***N*** | ***U***^+^ | **Tukey's or Holm's corrected *p*-value** | **Mean Difference^#^** | **Higher Group** |
| --- | --- | --- | --- | --- | --- | --- |
| GIP | **Seasons** | 156, 113 | 14155 | **0.000***** | 873 | **Wet** |
|  | Sexes | 128, 141 | 8714 | 0.626 | 115 | females |
|  | Ages | 56, 213 | 7102 | 0.084. | 394 | yearlings |
|  | MY, FY | 22, 34 | 502 | 0.084. | 662 | MY |
|  | **MY, MA** | 22, 106 | 1722 | **0.002**** | 935 | **MY** |
|  | **MY, FA** | 22, 107 | 1587 | **0.041*** | 658 | **MY** |
| GIS | **Seasons** | 156, 113 | 14031 | **0.000***** | 405 | **Wet** |
|  | Sexes | 128, 141 | 8743 | 0.655 | 29.7 | females |
|  | **Ages** | 56, 213 | 7142 | **0.043*** | 200 | **yearlings** |
| GIC | **Seasons** | 156, 113 | 14210 | **0.000***** | 6617 | **Wet** |
|  | Sexes | 128, 141 | 9468 | 0.483 | 1289 | males |
|  | **Ages** | 56, 213 | 7729 | **0.002**** | 2397 | **yearlings** |
|  | **MY, FA** | 22, 107 | 1678 | **0.003**** | 2561.13 | **MY** |
| FGM | Seasons | 151, 111 | 8147 | 0.701 | 971 | Dry |
|  | Sexes | 127, 135 | 9696 | 0.137 | 459 | males |
|  | Ages | 55, 207 | 4694 | 0.137 | 1128 | adults |
|  | MY, MA | 22, 105 | 767 | 0.054. | 1722 | MA |
|  | **WetM, WetF** | 70, 81 | 3735 | **0.005**** | 976 | **WetM** |
|  | WetF, DryF | 81, 54 | 1742 | 0.183 | 1604 | DryF |
| FPM4 | **Seasons** | 63, 49 |  | **0.020*** | 134 | **Wet** |
|  | Sexes | 24, 88 |  | 0.543 | 21.5 | males |
|  | Ages | 22, 90 |  | 0.228 | 36.6 | adults |
| FEM4 | **Seasons** | 63, 49 |  | **0.000***** | 1053 | **Dry** |
|  | Sexes | 24, 88 |  | 0.101 | 613 | males |
|  | Ages | 22, 90 |  | 0.302 | 197 | adults |
|  | **DryM, WetF** | 18, 56 |  | **0.000***** | 1123 | **DryM** |
|  | **WetF, DryF** | 56, 32 |  | **0.000***** | 1045 | **DryF** |
| FTMsqrt | Seasons | 45, 32 |  | 0.847 | 4.62 | Dry |

Significant tests (*p*< 0.05) are in bold.

For non-normal variables, interaction effect comparisons were only explored with Wilcoxon rank sum tests if preliminary explorations with ANOVA and Tukey HSD tests revealed a *p*<0.1. For normalized variables examined with Type III ANOVAs and Tukey HSD tests, only significant interaction comparisons (*p*<0.05) are listed here.

*N*'s are listed as males always first; then yearlings first; then Wet Season first.

^@^ MY=male yearlings; MA=male adults; FY=female yearlings; FA=female adults

^+^ Comparisons listed with a *U* test statistic were analyzed with Wilcoxon rank sum tests. *p* values for

these tests were adjusted for familywise error rates using the Holm's Bonferroni correction.

^#^ Mean differences are differences between non-transformed means for all variables. Units for mean

differences are eggs per gram of feces for GIP and GIS; oocysts per gram of feces for GIC; ng/g of

fecal dry weight for FGM and FPM; and pg/g of fecal dry weight for FPM.

. *p*<0.1; **p*<0.05; ***p*<0.01; ****p*<0.001

**Table K. Hormone assay sensitivities and detection limits.**

| **Assay** | **Assay Sensitivity** | **Minimum Detection Limit** | **Maximum Detection Limit** | **Intra-assay Coefficient of Variation** | **Inter-assay Coefficient of Variation** |
| --- | --- | --- | --- | --- | --- |
| Cortisol | 12.5ng/ml | 25ng/ml | 1000ng/ml | <4% | <9% |
| Progesterone | 0.2ng/ml | 0.3ng/ml | 90ng/ml | <5% | <10% |
| Estrogen | 0.6pg/ml | 5pg/ml | 750pg/ml | <5% | <10% |
| Testosterone | 0.05ng/ml | 0.1ng/ml | 20ng/ml | <8% | <12% |
