# Supplementary figures and images for: Seasonal Patterns of Hormones, Macroparasites, and Microparasites in Wild African Ungulates: The Interplay among Stress, Reproduction, and Disease
Source: PLoS One. 2015 Apr 15;10(4):e0120800. doi: 10.1371/journal.pone.0120800 (PMC4398380; doi:10.1371/journal.pone.0120800)

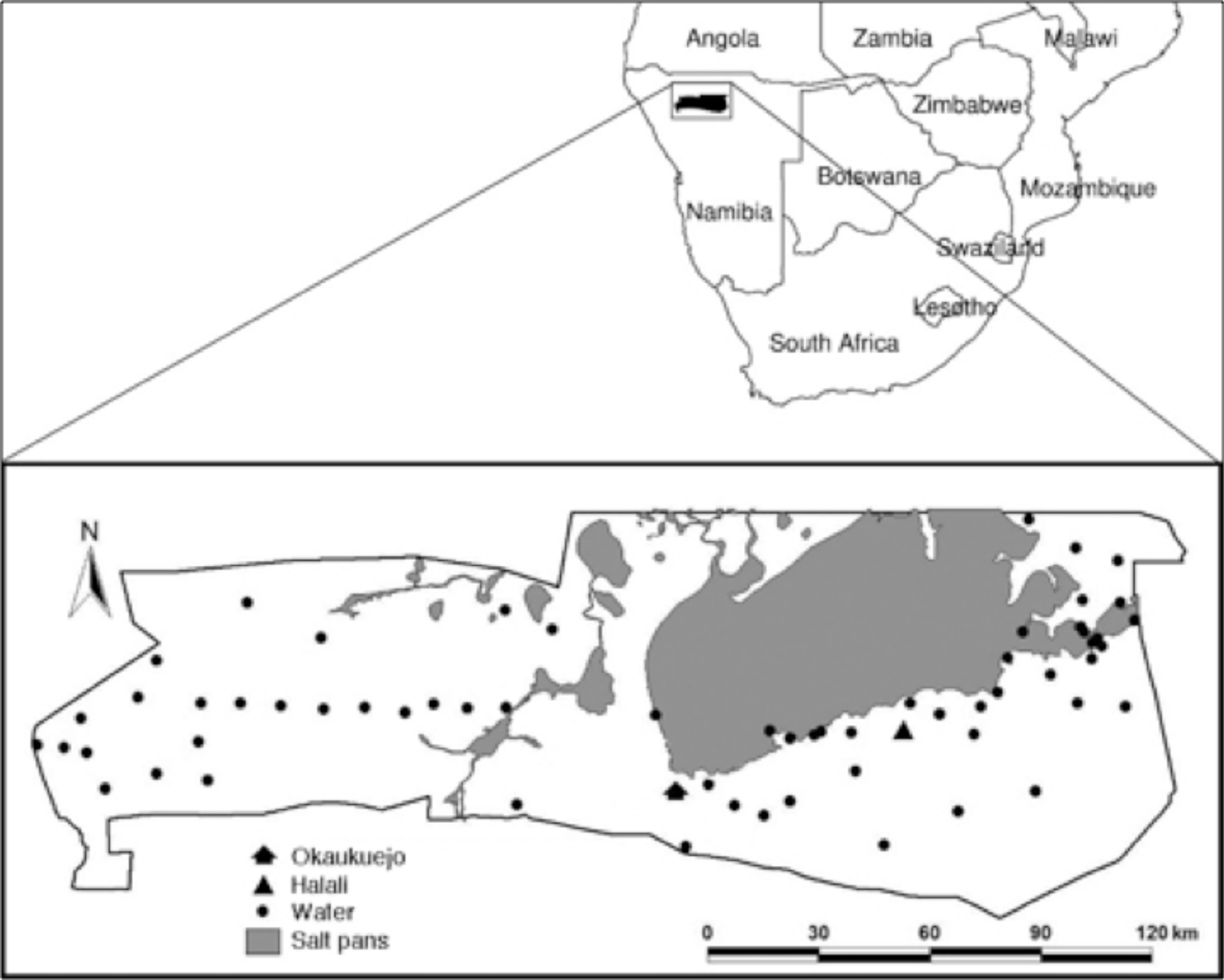

Supplement: S1 File — This file contains supplementary methods for laboratory protocols and statistical analyses, a map of the study region, and tables with information about animal captures, variables and abbreviations, maximal GEE and GLM models, and statistical results for seasonal and sex and age group comparisons. (ZIP) [file pone.0120800.s001.zip › FigA.tif]

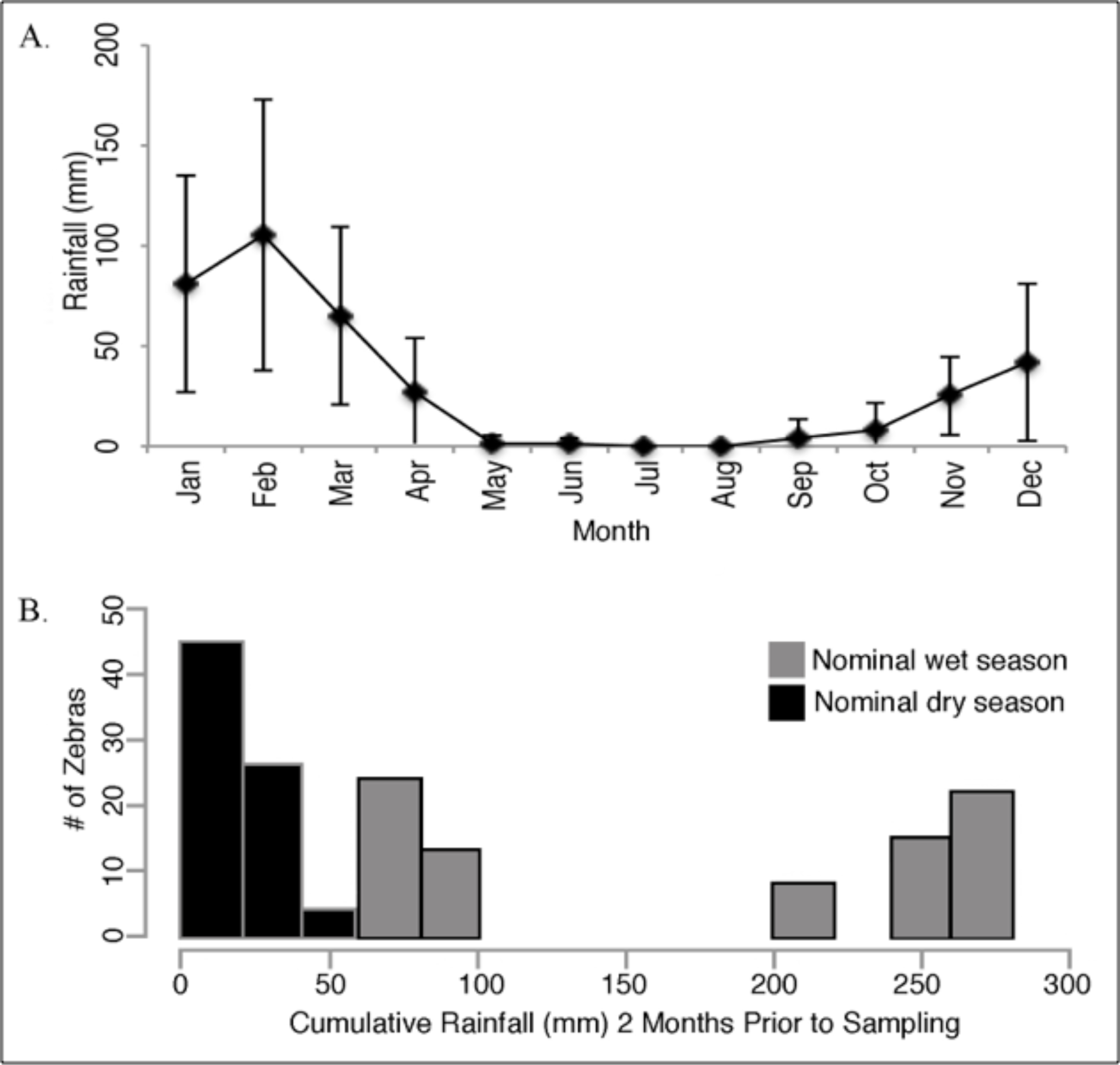

Supplement: S1 File — This file contains supplementary methods for laboratory protocols and statistical analyses, a map of the study region, and tables with information about animal captures, variables and abbreviations, maximal GEE and GLM models, and statistical results for seasonal and sex and age group comparisons. (ZIP) [file pone.0120800.s001.zip › FigB.tif]
